# Modeling and Simulation of Mass Transfer in Food Processing: Recent Advances in Governing Equations, Workflow, and Applications

**DOI:** 10.3390/foods15122084

**Published:** 2026-06-08

**Authors:** Sihui Chen, Zhou Qin, Tianxing Wang, Junjun Zhang, Roujia Zhang, Yucheng Zou, Jiyong Shi

**Affiliations:** School of Food Science and Engineering, Jiangsu University, Zhenjiang 212013, China; chensihui56@163.com (S.C.); 19599960979@163.com (Z.Q.); junjun_5457@ujs.edu.cn (J.Z.); 1000005191@ujs.edu.cn (R.Z.); yuchengzou@ujs.edu.cn (Y.Z.)

**Keywords:** food processing, mass transfer, modeling and simulation, governing equations, simulation workflow

## Abstract

Mass transfer is central to food processing but remains difficult to quantify because food materials are heterogeneous, multiphase, porous, biologically structured, and dynamically changing. Under these conditions, experiments alone cannot fully capture the spatiotemporal complexity of transport behavior, making modeling and simulation essential for mechanism interpretation, process prediction, and engineering optimization. Existing reviews mainly address specific operations or numerical methods, with limited synthesis of governing equations, simulation workflows, application implementation, and practical applicability. This review examines food mass transfer by linking coupled momentum, heat, and mass transfer laws with governing equation selection, simulation workflow, and representative food processing applications. Governing formulations for Fickian diffusion, conservation-based transport, heat–mass coupling, multicomponent transfer, Darcy-type porous-medium flow, and related model extensions are summarized, together with their assumptions, geometric applicability, and dimensionless criteria. A unified simulation workflow is then organized, covering transport type identification, governing equation and physical model selection, geometric representation, parameter determination, initial and boundary condition specifications, numerical method and simulation tool selection, numerical implementation, validation, and transferability assessment. Representative applications are discussed for drying, heat–mass coupled processes, multicomponent transfer, transport in porous foods, and redistribution in multi-ingredient or multilayer foods. Overall, future progress requires more integrated, structure-aware, experimentally validated, transferable, and application-oriented simulation frameworks.

## 1. Introduction

The increasing demand for high-quality, safe, and nutritious foods has promoted the continuous development of advanced food processing technologies. In drying [1], curing [2], rehydration [3], and thermal treatment [4,5], product transformation is governed not only by mass transfer, such as moisture migration, solute diffusion, and gas transport, but also by momentum and heat transfer in the surrounding medium and within the food matrix. External airflow or oil flow controls convective exchange at the product surface, heat transfer determines temperature evolution and phase change, and mass transfer governs the redistribution or removal of water, solutes, and gases. These coupled transport phenomena directly affect product quality, texture development, storage stability, process efficiency, and energy utilization [6,7]. However, food materials are typically heterogeneous, multiphase, porous, and structurally dynamic [1,8,9]. Their transport behavior is therefore often nonlinear, spatially nonuniform, and strongly coupled with changes in microstructure and physicochemical properties. For biological tissues, this heterogeneity also includes cell membranes and cell walls, whose integrity can strongly affect internal resistance to water and solute migration [10,11]. Under such conditions, a quantitative description of mass transfer remains a central challenge in food engineering.

Conducting a direct experimental investigation of internal mass transfer is difficult. Conventional approaches often rely on destructive sampling and pointwise measurements, which are labor-intensive, time-consuming, and inadequate for capturing the spatiotemporal evolution of transport processes [12]. Recent advances in nondestructive and in situ characterization methods, such as nuclear magnetic resonance (NMR), X-ray computed tomography (CT), and hyperspectral imaging (HSI), have greatly improved the ability to visualize internal structural changes and component distributions during processing [13,14,15]. Nevertheless, characterization techniques mainly provide observational evidence and empirical data. Mechanistic interpretation and quantitative prediction still depend on physically based models. For this reason, modeling and numerical simulation are increasingly used in food mass transfer research. By integrating governing equations, material properties, and boundary conditions, simulation can be used to predict concentration fields, diffusion pathways, and flow behavior, thereby supporting process analysis, virtual experimentation, and optimization [9,16].

Despite the growing body of literature, existing reviews remain largely fragmented. Many are organized around specific processing operations [17,18,19,20], while others focus on particular experimental or numerical methods [9,21], without clearly linking governing equations, simulation workflow designs, and representative food processing applications. This fragmentation also limits the use of simulation results for interpreting food quality changes and guiding process optimization across different processing conditions. More importantly, limited attention has been paid to how experimental characterization supports model construction, parameter determination, and validation within a unified simulation framework. Accordingly, this review links food mass transfer mechanisms with equations, workflows, and applications.

This review first summarizes the governing equations commonly used to describe diffusion, heat–mass coupled transport, multicomponent transfer, and porous-medium transport in food materials. It then organizes a unified simulation workflow for mass transfer, including transport type identification, governing equation and physical model selection, geometric representation, parameter determination, initial and boundary condition specification, numerical method and simulation tool selection, numerical implementation, and validation. Selected representative applications are subsequently discussed to show how these workflow decisions are implemented in drying and dehydration, heat–mass coupled processes, multicomponent solute transfer, transport in porous foods, and moisture redistribution in multi-ingredient or multilayer foods.

## 2. Governing Equations and Physical Basis of Coupled Transport in Food Processing

Transport simulation in food processing requires a clear distinction between the physical laws governing momentum, heat, and mass transfer [21]. In drying, frying, baking, curing, rehydration, and osmotic dehydration, moisture or solute migration rarely occurs as an isolated diffusion process. Instead, it is often coupled with external airflow or liquid flow, convective heat exchange at the product surface, internal heat conduction, phase change, pressure-driven flow, and structural evolution [1,22]. Therefore, the selection of governing equations should begin with the dominant transport mechanism, the degree of coupling among transport processes, and the geometric assumptions used to simplify the food material [23].

Momentum transfer is relevant when airflow, oil flow, vapor movement, or pressure-driven liquid flow affects external exchange or internal migration [1,24]. Heat transfer determines the temperature field that drives evaporation, thermal softening, phase transition, and temperature-dependent diffusivity [21]. Mass transfer describes the redistribution or removal of water, solutes, oil, and gases within the food matrix [25]. Before discussing specific mass transfer formulations, the fundamental phenomenological laws, geometric applicability, and dimensionless criteria are summarized to provide a physical basis for model selection.

### 2.1. Fundamental Transport Laws and Dimensionless Criteria

Food processing operations involve several coupled transport laws rather than a single mass-diffusion equation [21,26]. For momentum transfer, Newton’s law of viscosity provides the local relationship between shear stress and velocity gradient and is relevant to airflow, oil flow, vapor movement, and boundary-layer development near the food surface. For heat transfer, Fourier’s law describes internal heat conduction, whereas Newton’s law of cooling is commonly used as a convective boundary condition between the food surface and the surrounding medium. For mass transfer, Fick’s laws describe diffusion driven by concentration or moisture gradients and are widely used as the starting point for modeling drying, soaking, curing, and rehydration [23].

These laws are not used independently in most thermal food processes. In drying, airflow affects the external heat and mass transfer coefficients, heat transfer controls the temperature field and evaporation rate, and mass diffusion governs internal moisture redistribution. In frying and baking, heat and mass transfer are further coupled with phase change, crust formation, oil uptake, and structural deformation [1,24,27]. Therefore, the equations listed in Table 1 should be understood as complementary components of coupled transport models rather than isolated equations.

The simplified use of these equations also depends on the choice of characteristic length, flow regime, and relative importance of internal and external resistances [23,26,30]. These aspects are commonly evaluated using dimensionless numbers. In food drying and related processes, dimensionless numbers connect momentum, heat, and mass transfer by comparing inertial, viscous, conductive, convective, and diffusive effects. They are also useful for selecting model assumptions, interpreting boundary conditions, and judging whether lumped, one-dimensional, or multidimensional models are appropriate. Table 2 summarizes the main dimensionless numbers used to describe coupled transport in food processing simulations.

### 2.2. Fick’s Laws

Fick’s laws are the most widely used basis for describing mass diffusion in food systems [23,31]. They are especially suitable for processes in which mass transfer is mainly driven by concentration gradients and convection is negligible. In the drying of fruits and vegetables, for example, Fick-based models are often used to estimate the migration of internal moisture toward the surface and to predict the time required to reach the target moisture content [17]. Owing to their simplicity and clear physical meaning, Fick-based models also provide the foundation for more complex transport formulations.

#### 2.2.1. Fick’s First Law

Fick’s first law describes steady-state diffusion that is driven by a concentration gradient [32]. In food systems, it is mainly useful for interpreting diffusion flux under steady or quasi-steady conditions. It can be expressed by Equation (1):

(1) $$J=-D\frac{\partial C}{\partial x}$$

where J is the diffusion flux (mol·m^−2^·s^−1^), D is the diffusion coefficient (m^2^·s^−1^), $\frac{\partial C}{\partial x}$ is the concentration gradient (mol·m^−4^), and C is the concentration of the diffusing component (mol·m^−3^).

#### 2.2.2. Fick’s Second Law

Fick’s second law extends diffusion analysis to transient conditions by describing how concentration changes with time during diffusion [33]. It can be expressed by Equation (2):

(2) $$\frac{\partial C}{\partial t}=D\frac{\partial^{2}C}{\partial x^{2}}$$

where $\frac{\partial C}{\partial t}$ is the rate of change in concentration C with time t, D is the diffusion coefficient (m^2^·s^−1^), and $\frac{\partial^{2}C}{\partial x^{2}}$ is the second derivative of concentration C with respect to the space coordinate x.

For multidimensional transport with anisotropic or direction-dependent properties, the equation can be written as Equation (3):

(3) $$\frac{\partial C}{\partial t}=\frac{\partial }{\partial x}(D_{x}\frac{\partial C}{\partial x})+\frac{\partial }{\partial y}(D_{y}\frac{\partial C}{\partial y})+\frac{\partial }{\partial z}(D_{z}\frac{\partial C}{\partial z})$$

where $D_{x}$, $D_{y}$, and $D_{z}$ represent the effective diffusivities in the three coordinate directions.

Because most food processes are transient, Fick’s second law is commonly used to predict time-dependent concentration or moisture profiles in drying, soaking, curing, and dehydration [34,35]. However, the simplified one-dimensional form of this equation should be used only when transport in one direction dominates [34]. This condition is usually satisfied for an infinite slab or plate, where one dimension is much smaller than the other two, or for an infinite cylinder, where radial transport dominates and axial gradients can be neglected [36]. For finite solids with comparable dimensions, such as cubes, thick slices, fries, bakery products, or irregular food pieces, resistance to heat and mass transfer may be comparable in more than one direction. In such cases, two- or three-dimensional formulations of Fick’s second law, or numerical methods such as FEM-, FVM-, or CFD-based approaches, are required to represent multidirectional transport more accurately.

### 2.3. Mass Conservation Equations

Although Fick’s second law can be derived from mass conservation under diffusion-dominated conditions, more general mass conservation equations are required when convection, flow, or source terms must be considered. In food processing, such conservation-based formulations are useful when the transported component is governed not only by molecular diffusion but also by phase movement, interfacial exchange, phase transition, or local generation and consumption, as shown in recent coupled transport simulations of pasta drying, grain-pile drying, and microwave drying systems [37,38,39]. Compared with simple diffusion models, conservation-based formulations offer greater flexibility for representing realistic processing conditions. These formulations are particularly relevant when moisture loss, vapor migration, oil uptake, or local phase change affects product quality during thermal processing. A general form for a given component (A) can be written as Equations (4) and (5) [37,40]:

(4) $$\frac{\partial C_{A}}{\partial t}+\nabla \cdot (C_{A}v_{A}+J_{A})=R_{A}$$

(5) $$J_{A}=-D_{A}\nabla C_{A}$$

where $C_{A}$ is the concentration of component A(mol·m^−3^), $v_{A}$ is the phase velocity of component A(m·s^−1^), $J_{A}$ is the diffusion flux of component A(mol·m^−2^·s^−1^), $R_{A}$ is the reaction/sink term of component A (mol·m^−3^·s^−1^) ($R_{A}$ > 0 is generation and $R_{A}$ < 0 is consumption), and $D_{A}$ is the diffusion coefficient of component A (m^2^·s^−1^).

### 2.4. Maxwell–Stefan Equations

Food systems are often multicomponent in nature, and interactions among water, solutes, and other components can strongly affect transport behavior. In such cases, the Maxwell–Stefan equation can provide a more rigorous framework when species interactions are central because it accounts for frictional interactions among species in multicomponent systems [29,41,42]. This is particularly relevant to osmotic dehydration and compound marination, where water and multiple solutes migrate simultaneously and competitively [43,44]. Therefore, the Maxwell–Stefan framework is valuable when coupled transport between components cannot be adequately simplified as effective binary diffusion. This framework is especially relevant to salt, sugar, and water redistribution, which affects flavor uniformity, texture, and product stability. A general Maxwell–Stefan formulation can be expressed as Equation (6):

(6) $$-\nabla x_{i}=\sum_{j\neq i}\frac{x_{j}N_{i}-x_{i}N_{j}}{cD_{ij}}$$

where $x_{i}$ is the mole fraction of the *i*-th component, $N_{i}$ is the molar flux of component *i* (mol·m^−2^·s^−1^), c is the total molar concentration (mol·m^−3^), and $D_{ij}$ is the Maxwell–Stefan diffusivity between components *i* and *j* (m^2^·s^−1^).

### 2.5. Darcy’s Law

Many food materials, such as bread, cakes, fruits, vegetables, and dried products, contain porous structures. In these systems, Darcy’s law provides the basic framework for describing pressure-driven fluid flow through porous media [8,45]. It can be expressed by Equation (7):

(7) $$q=-\frac{K}{\mu }\nabla p$$

where q is the apparent flux (m·s^−1^), K is the permeability of porous media (m^2^), μ is the dynamic viscosity (Pa·s), and ∇p is the pressure gradient (Pa·m^−1^).

By incorporating permeability and pressure-driven flow, Darcy-based formulations are useful for analyzing moisture penetration, liquid expulsion, and gas transport in porous foods [45,46,47]. These models are especially relevant to drying, cooling, and rehydration, where pore structure strongly influences transport efficiency. However, when pore-scale effects, viscous shear, or multiphase interactions become significant, Darcy’s law alone may be insufficient and may need to be coupled with additional transport equations.

### 2.6. Key Transport Parameters

Transport parameters determine both the rate and pathway of mass transfer in food materials. Diffusion coefficients are central to mass transfer processes driven by concentration gradients, while effective diffusivity is often more meaningful in heterogeneous foods because it incorporates the effects of porosity, tortuosity, confinement, shrinkage, and thermal activation [48]. In intact plant and muscle tissues, water and solutes do not migrate through a homogeneous continuum. Their movement can be constrained by cell membranes, cell walls, intercellular spaces, vascular bundles, connective-tissue frameworks, and pore networks [49,50]. Cell membranes are particularly important because they separate intracellular and extracellular compartments and may form a significant internal resistance to water and solute migration [10].

Because these structural effects are usually embedded in fitted transport parameters, effective diffusivity should be interpreted as an apparent coefficient influenced by membrane permeability, tissue integrity, and processing history rather than as a universal material constant [51]. This does not mean that the assumption of constant effective diffusivity is always inappropriate. It may be acceptable when the process is conducted within a narrow temperature and moisture range, when shrinkage and microstructural changes are limited, when the food matrix can be approximated as homogeneous, and when the modeling objective is restricted to fitting overall drying, soaking, or moisture-uptake curves rather than predicting internal spatial gradients [52]. Constant *D*_eff_ may also be reasonable for preliminary comparison among treatments or for materials whose cellular structure has already been largely disrupted, such as powders, ground foods, or mechanically homogenized matrices. In these cases, the fitted diffusivity should still be interpreted as an apparent parameter valid only within the calibrated material, geometry, and operating range.

For porous foods, porosity, tortuosity, and permeability are also important because they control the accessibility, continuity, and resistance of transport pathways [8,53]. Permeability is particularly relevant for Darcy-based models, as it characterizes the ease of fluid movement through pore networks under a pressure gradient [54]. In practical food systems, these parameters are often structure-dependent and may change during swelling, shrinkage, pore collapse, or phase transition [55]. External mass transfer coefficients may also be required when internal diffusion is coupled with surface exchange [56]. Parameter estimation affects both numerical accuracy and the physical interpretability of simulated transport pathways.

Pretreatments can substantially modify these transport parameters. Blanching can soften tissues, disrupt membrane integrity, and reduce internal resistance to moisture movement, while pulsed electric field treatment can induce electroporation and increase cell membrane permeability [57,58]. Freezing, cutting, grinding, and other mechanical or thermal operations may also alter the continuity of transport pathways. This issue is less critical for powdered, ground, or mechanically disrupted foods, where the original cellular compartmentalization has already been largely reduced or destroyed. By contrast, for intact fruits, vegetables, tubers, grains, and muscle foods, the biological state of the tissue should be considered during parameter determination, model selection, and validation [59]. When pretreatment changes membrane permeability or tissue structure, diffusivity, permeability, and surface or internal transfer coefficients should ideally be treated as pretreatment-dependent or state-dependent quantities rather than universal material constants [51,59].

The equations discussed above differ not only in mathematical form but also in their assumptions, applicable transport conditions, and predictive limitations [21]. For this reason, the selection of a governing equation should not be based only on familiarity or simplicity, but on the dominant transport mechanism, material structure, availability of parameters, and validation objective. Table 3 summarizes the main governing equation frameworks used in food mass transfer simulation and compares their transport conditions, assumptions, representative applications, advantages, and limitations.

As shown in Table 3, governing equation selection is a process-specific decision. Fick-based models are useful for diffusion-dominated systems and preliminary kinetic fitting, whereas conservation-based, coupled heat–mass, Maxwell–Stefan, Darcy, or porous-medium formulations are needed when flow, phase changes, multicomponent interactions, pressure gradients, or pore-scale structure become important. Therefore, the governing equation should be selected together with geometry, parameter representation, boundary conditions, numerical methods, and validation strategies. This provides the basis for the unified simulation workflow discussed in the next section.

## 3. A Unified Simulation Workflow for Mass Transfer in Food Processing

Mass transfer in food processing is usually transient, spatially heterogeneous, and closely coupled with changes in food structure, composition, and physicochemical properties. As a result, numerical simulation is not only used to calculate moisture, solute, or gas transport, but also to organize experimental observations into a physically interpretable framework. For food science and technology applications, such a workflow is also important because simulated fields can be linked to quality-related responses such as moisture uniformity, solute distribution, texture formation, and processing efficiency.

**Figure 1:**
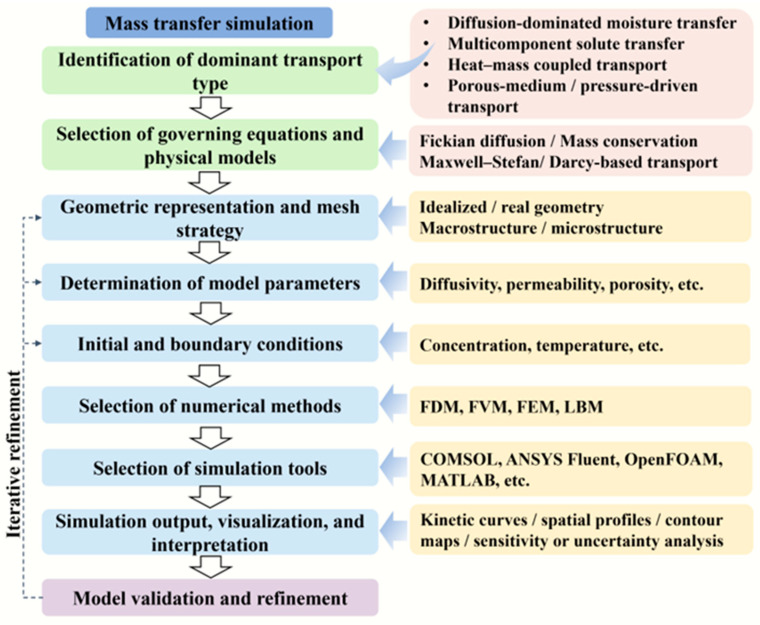
Unified simulation workflow for mass transfer in food processing.

As illustrated in Figure 1, a unified simulation workflow for mass transfer begins with the identification of the dominant transport type, such as diffusion-dominated moisture transfer, multicomponent solute transfer, heat–mass coupled transport, or porous-medium transport. On this basis, appropriate governing equations and physical models are selected, followed by geometric representation and a mesh strategy, determination of the model parameters, and specification of the initial and boundary conditions. The model is then solved using suitable numerical methods and simulation tools, and the results are interpreted through kinetic curves, spatial distributions, quantitative indicators, and comparison with analytical or experimental data. Although these steps are presented in a logical sequence, they are not strictly linear. In practice, parameter determination, boundary condition setting, mesh refinement, and validation often require repeated adjustments. A unified simulation workflow is therefore necessary to improve the transparency, repeatability, and reliability of mass transfer simulation in food processing. Importantly, validation often leads to iterative refinement of geometric representation, parameter values, and boundary condition specification rather than a one-step confirmation of model performance.

### 3.1. Identification of Dominant Transport Type

The identification of the dominant mass transfer mechanism is the starting point of the simulation, as it defines how the transport problem should be mathematically represented. In food systems, transport may be driven by concentration gradients, fluid flow, multicomponent interactions, or pressure gradients within porous structures [1,15,60,61]. In many practical processes, such as drying, frying, and marinating, these mechanisms coexist and interact. A key limitation in current practice is the tendency to classify transport behavior based on simplified assumptions rather than quantitative analysis. For example, processes are often treated as diffusion-dominated transport without evaluating the contribution of convection or structural constraints [62,63]. This may lead to inappropriate model selection and misinterpretation of transport behavior.

Dominant transport identification is more defensible when process characteristics are supported by dimensionless analysis or a time-scale comparison [63]. Such approaches help distinguish whether diffusion, convection, or structural resistance governs the process. Importantly, this step should not be treated as a one-time classification, but as a hypothesis that may need to be revised during model validation and refinement.

### 3.2. Selection of Governing Equations and Physical Models

Following the identification of the transport mechanism, appropriate governing equations must be selected to represent the underlying physics. Fick-based diffusion models are commonly used when concentration gradients dominate and when convection is negligible [31,62]. In contrast, conservation-based formulations are required when transport is coupled with flow, phase change, or source terms [1,61]. For multicomponent systems, Maxwell–Stefan equations provide a more rigorous framework, while porous-medium transport often requires Darcy-based models [54,64].

Despite the availability of these frameworks, model selection in food mass transfer studies is frequently driven by simplicity rather than physical consistency. In particular, the widespread use of Fickian models without verifying their underlying assumptions remains a major limitation [31,62]. Such models implicitly assume homogeneous structure, constant parameters, and negligible coupling effects—conditions that are rarely satisfied in real food systems.

Therefore, model selection should follow a hierarchical and decision-oriented logic. Simplified models may be appropriate for preliminary analysis or when transport mechanisms are clearly dominated by a single driving force [31]. However, when structural heterogeneity, multiphase interactions, or strong coupling effects become significant, more comprehensive formulations are required. Importantly, increasing model complexity should not be an end in itself. The selected model should balance physical realism, parameter availability, and computational feasibility, ensuring that added complexity translates into meaningful improvement in predictive capability.

Recent drying simulations have increasingly moved beyond constant-diffusivity Fickian descriptions toward formulations that incorporate state-dependent parameters, coupled heat and mass transfer, shrinkage, porous-medium transport, external flow fields, and structure-informed geometries [21,51]. These developments do not necessarily represent entirely new physical laws; rather, they extend classical conservation equations by introducing more realistic material functions, boundary conditions, source terms, moving domains, or multiscale structural information. Table 4 summarizes the representative advances in governing formulations and modeling strategies for food drying simulations. The purpose of this table is not to rank model complexity but to clarify how recent studies have modified classical equations to address specific limitations in drying-process simulation.

As shown in Table 4, recent advances in drying simulation are mainly reflected in the way classical equations are extended, coupled, parameterized, or implemented in realistic geometries. Therefore, the workflow proposed in this section should not be interpreted as a fixed sequence of operations. Instead, the selected formulation determines the required level of geometric representation, parameter determination, boundary condition specification, numerical method selection, and validation. For example, a constant-diffusivity Fickian model may require only a simple geometry and kinetic validation, whereas a shrinkage-coupled or CFD-coupled model requires more detailed geometry, additional parameters, and stronger validation evidence [26,52,74].

### 3.3. Geometric Representation and Mesh Strategy

**Figure 2:**
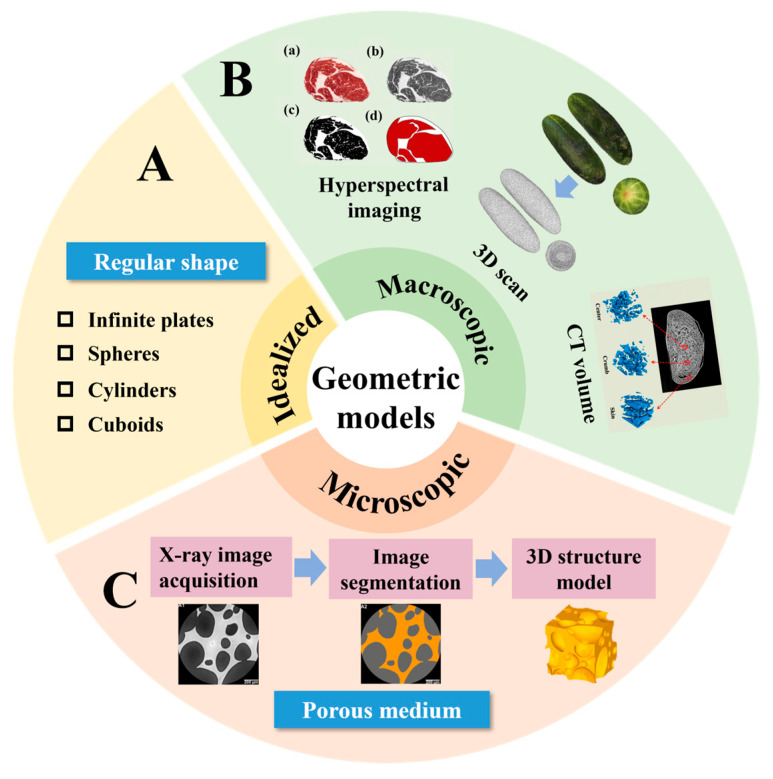
Geometric representation strategies for food materials: (**A**) idealized geometry, (**B**) macroscopic image-based representation, and (**C**) microscopic pore-scale representation. Adapted from Refs. [78,79,80,81].

Geometric representation governs the translation of food microstructure into a computational domain, directly influencing simulation fidelity and cost [75]. The choice is rarely a simple trade-off between abstraction and complexity; rather, it involves selecting a level of structural detail justified by the modeling objective. For foods with regular shapes, simplified geometries such as slabs, cylinders, spheres, or cuboids are often adopted as idealized domains (Figure 2A) [76]. The choice of simplified geometry should be based on directional transport resistance rather than geometric convenience alone [21,51]. One-dimensional diffusion models are appropriate only when one dimension controls the dominant transport path, as in thin slabs or sufficiently long cylinders. When the product dimensions are comparable, heat and mass transfer resistance may be significant along several directions, and the final moisture or temperature field is affected by multidirectional transport. Therefore, cuboids, thick slices, fries, filled products, and irregular foods generally require 2D or 3D numerical domains if spatial gradients are important [61,67]. These idealized geometries are computationally efficient and sufficient for bulk transport simulations under the assumption of homogeneity. However, real food systems often exhibit irregular external morphology, heterogeneous internal composition, and anisotropic transport pathways [77]. Under such conditions, image-based reconstruction from techniques such as HSI [78], 3D scanning [79], or X-ray CT [80] can provide more realistic geometric descriptions and better preserve structural features relevant to mass transfer, including irregular boundaries, tissue heterogeneity, and directional transport paths (Figure 2B). The gain in realism, however, is accompanied by greater demands in geometric preprocessing, parameter specification, and numerical computation.

For porous foods, geometric representation may need to extend beyond external shape toward internal pore architecture. In such cases, pore-scale or hierarchical representations based on X-ray to obtain pore connectivity and void structure can offer additional insight into liquid and gas transport that cannot be captured by macroscopic idealization alone (Figure 2C) [81]. This suggests that geometric representation in food mass transfer simulation is best treated as a problem-dependent balance rather than a simple progression toward maximal realism. Simplified geometry is appropriate when overall trends are sufficient, whereas structure-based or multiscale representation becomes more valuable when internal heterogeneity is expected to influence transport behavior [62,77].

Mesh generation is closely tied to this choice because it governs numerical accuracy and solution stability. Structured meshes are generally suitable for regular domains, whereas irregular or reconstructed geometries more often require unstructured or hybrid meshes [82]. In addition, local refinement is usually more important than uniform mesh densification, especially in regions with steep gradients, strong interfacial transfer, or local deformation. For processes such as drying and frying, where shrinkage or shape change may occur during moisture loss, adaptive or dynamic meshing may further be needed to maintain geometric consistency during simulation [83,84].

### 3.4. Determination of Model Parameters

Model parameters determine the diffusion rates, flow resistance, local pathways, and simulated concentration or moisture fields. Key parameters include diffusion coefficients, effective diffusivity, porosity, tortuosity, and permeability, depending on the selected model [54,62]. A major challenge in food systems is that these parameters are not constant but depend on temperature, moisture content, composition, and structural evolution [52,54]. For biological tissues, parameter determination should also consider the integrity of cell membranes and the pretreatment history of the material. Blanching, pulsed electric field treatment, freezing, cutting, and mechanical disruption may change membrane permeability and tissue connectivity, thereby altering the apparent effective diffusivity, permeability, and surface or internal transport resistance [58,59,85]. Nevertheless, many studies treat them as fixed values, which may oversimplify the actual transport behavior and lead to discrepancies between simulation and experiment [64].

In addition, parameter determination often relies on inverse modeling, where parameters are adjusted to fit experimental data [86]. Such problems are frequently ill-posed, meaning that multiple parameter sets may produce similar simulation results [75]. This raises concerns regarding parameter identifiability and the physical meaning of fitted values. More importantly, inverse-estimated parameters are often conditional rather than universal. A diffusivity, permeability, or transfer coefficient fitted from one material, maturity stage, geometry, pretreatment, or equipment design may not remain valid when the boundary conditions or product properties change [52,87]. Therefore, parameter fitting should be distinguished from model validation. A model that reproduces the dataset used for parameter estimation cannot be assumed to have predictive capability unless it is tested against independent conditions, materials, or equipment configurations [51,87].

To address these issues, parameter determination should be viewed as a process of selection, representation, and validation rather than simple data input. Where possible, parameters should be measured directly or supported by independent experiments [60]. Sensitivity analysis and uncertainty quantification should be incorporated to evaluate the robustness of model predictions [88]. Furthermore, representing parameters as functions of state variables, rather than constants, can improve the realism of simulations under dynamic conditions.

### 3.5. Initial and Boundary Conditions

Initial and boundary conditions define the starting field, surface exchange, and driving forces imposed on the food domain. Initial conditions describe the starting distribution of variables such as moisture content or solute concentration, while boundary conditions represent exchange mechanisms at the interface [75,89].

In practice, these conditions are often simplified due to limited experimental data [75]. For example, constant surface conditions or simplified flux boundaries are frequently assumed in drying models [75]. Such assumptions may not accurately reflect real processing environments, where boundary conditions can vary spatially and temporally.

Therefore, boundary conditions should be selected based on physical mechanisms rather than numerical convenience [90]. Whenever possible, they should be informed by experimental measurements or justified by process analysis. Their influence on simulation results should be critically evaluated, as inappropriate boundary conditions may lead to significant errors even when the governing equations are correct [91].

### 3.6. Selection of Numerical Methods

The selection of numerical methods in food mass transfer simulation should be driven by the transport characteristics of the process rather than by numerical convenience alone. In most food applications, the central issue is whether the problem is dominated by simple diffusion or involves stronger geometric complexity, flow participation, and multiphysics coupling. For relatively simple diffusion-dominated systems with regular domains, the finite difference method (FDM) is often sufficient because it can describe the overall concentration evolution with relatively low computational cost [73]. Once convection, local conservation, and transport heterogeneity become important, however, the finite volume method (FVM) is usually more suitable. This is why it is widely used in drying, frying, and cooling simulations implemented in CFD frameworks such as ANSYS Fluent and OpenFOAM [19,26,92,93].

When food materials exhibit irregular geometry, anisotropy, deformation, or strong coupling among heat, mass, and structural responses, the finite element method (FEM) is generally more advantageous because of its flexibility in handling complex domains and coupled field behavior, as illustrated in simulations of drying-induced deformation in potato slices [84]. By comparison, the lattice Boltzmann method (LBM) is still less commonly used, but it is increasingly relevant for pore-scale and structurally heterogeneous transport, particularly in porous drying, precooling, and freezing [39,94,95,96,97]. Overall, these methods should be viewed as problem-dependent tools rather than competing choices: FDM is mainly appropriate for simplified diffusion analysis, FVM for conservation-based coupled transport with flow, FEM for complex multiphysics systems, and LBM for microstructure-informed transport problems.

### 3.7. Selection of Simulation Tools

Simulation tools influence how the selected physical model is implemented and therefore influence the feasibility, reproducibility, and credibility of mass transfer simulation [98,99,100]. As summarized in Table 5, commonly reported simulation environments such as COMSOL Multiphysics, ANSYS Fluent, OpenFOAM, and MATLAB differ in solver structure, available physics modules, flexibility for custom equations, and suitability for coupled transport problems or complex geometries. Therefore, software selection should not be treated as a post-computational choice. Instead, it should be aligned with the transport mechanism, degree of multiphysics coupling, geometric complexity, parameter availability, and available computational expertise. In this context, Table 5 is intended not to rank simulation platforms but to show how different tools can be matched with governing equations, physical models, and representative food mass transfer applications.

In practical terms, these simulation tools are more commonly used for research, product development, equipment design, process troubleshooting, and offline optimization than for routine production control [26,106]. Occasional industrially relevant applications include CFD-assisted analysis of airflow distribution in dryers or ovens, evaluation of heat and mass transfer uniformity in drying or baking chambers, and offline comparison of operating conditions before pilot-scale trials [107,108]. However, such use is still different from routine industrial implementation because the models often require case-specific geometry, material parameters, boundary conditions, and validation [26].

### 3.8. Simulation Output, Visualization, and Interpretation

**Figure 3:**
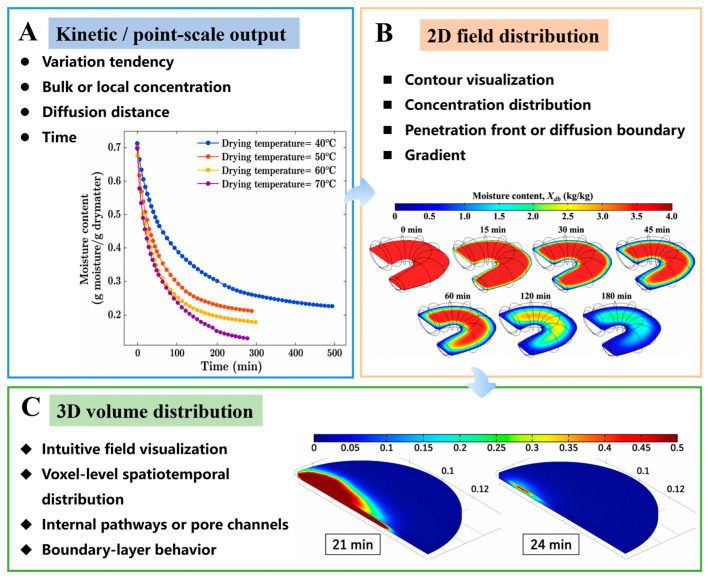
Typical forms of simulation output for food mass transfer studies: (**A**) kinetic or point-scale outputs, (**B**) two-dimensional field distributions, and (**C**) three-dimensional volume distributions. Adapted from Refs. [70,105,109].

Simulation results can be presented in multiple forms, including kinetic or point-scale outputs (Figure 3A), two-dimensional field distributions (Figure 3B), and three-dimensional volume distributions (Figure 3C). Kinetic or point-scale outputs are useful for tracking temporal changes in concentration or moisture content [105], whereas two-dimensional field maps reveal cross-sectional gradients and spatial heterogeneity [70]. Three-dimensional volume visualization provides a more intuitive representation of the overall transport field and is especially valuable for interpreting internal pathways, pore-channel effects, and boundary-layer behavior [109]. These different forms of output also determine what can be directly compared during validation, ranging from bulk kinetics to spatial field distributions.

Unlike point-based measurements, simulation can resolve time-dependent concentration or moisture fields across the domain. In numerical simulation platforms such as ANSYS and COMSOL Multiphysics, transport processes can also be displayed dynamically through animations, streamlines, slices, and volume-rendering maps. For example, the simulated diffusion of NaCl in steak clearly revealed nonuniform migration caused by the coexistence of muscle, fat, and connective tissue [60]. Such visualization is not only helpful for mechanism interpretation but also valuable for identifying local resistance, assessing uniformity, and determining process duration. More importantly, simulation output should not be regarded merely as a presentation tool. Proper visualization can help locate regions with steep gradients for local mesh refinement, support parameter calibration by comparing field evolution with imaging data, identify preferential pathways or retention zones in porous structures, and guide the selection of sampling or monitoring points. Therefore, the interpretation of simulation output should be linked directly to model refinement and process improvement.

### 3.9. Model Validation and Refinement

Model validation and refinement are indispensable for ensuring the reliability and practical value of the simulation results. In food mass transfer studies, validation is typically performed by comparing simulation outputs with experimental measurements and evaluating the agreement using statistical indices, error analysis, and key transport parameters such as effective diffusivity (*D*_eff_) [110,111]. In addition, different numerical methods may be used to solve the same problem, and the consistency of their results can be compared. In addition to direct experimental comparison, benchmark solutions and cross-method comparisons can be used to examine numerical stability, solver accuracy, and implementation consistency, particularly for simplified diffusion problems or newly developed numerical schemes [112].

Validation should be selected according to the type of simulation output. For kinetic or point-scale predictions, simulated moisture content, solute concentration, mass loss, or water uptake can be compared with experimental curves or fitted transport parameters. For spatially resolved simulations, field-level validation is more important because the model is expected to reproduce not only average changes but also internal gradients and nonuniform distributions. In this context, imaging methods can provide independent evidence for comparing predicted and observed concentration or moisture fields. For example, hyperspectral imaging has been combined with finite element analysis to validate the spatial distribution of sucrose during beef marination, thereby linking experimental visualization with model prediction [15]. Image-assisted monitoring has also been coupled with mass transfer simulation to evaluate moisture diffusion during soybean rehydration [113].

**Figure 4:**
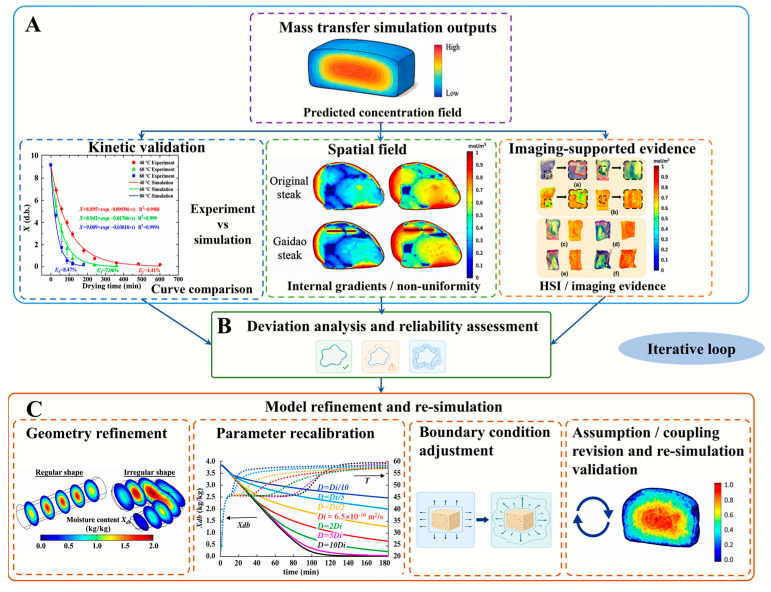
Validation-driven refinement of mass transfer simulation models in food processing: (**A**) mass transfer simulation outputs, (**B**) deviation analysis and reliability assessment, and (**C**) model refinement and re-simulation. Adapted from Refs. [70,78,114].

These examples indicate that effective validation in food mass transfer research often requires the integration of compositional measurement, imaging evidence, and numerical predictions rather than reliance on a single endpoint metric. As summarized in Figure 4, model reliability can be assessed through kinetic comparison, spatial field validation, and imaging-supported evidence. When deviations or uncertainties are identified, the model should be refined by recalibrating key parameters, revising modeling assumptions, improving geometric representation, specifying initial and boundary conditions more accurately, and verifying complex multiphysics couplings step by step. Thus, validation is better treated as an iterative process that links reliability assessment with model refinement and re-simulation. It should also be noted that imaging-based validation using MRI, CT, or similar high-cost techniques is mainly valuable for research-scale model construction and field-level validation but is rarely used as a routine industrial tool because of equipment costs, measurement times, sample handling requirements, and limited compatibility with continuous production environments.

Model validation should not be equated with fitting the same experimental data used for parameter estimation. In many food mass transfer studies, effective diffusivity, permeability, or boundary coefficients are inversely estimated from drying, hydration, salting, or frying experiments [52,115]. Such fitting can demonstrate descriptive adequacy under a specific condition, but it does not necessarily prove that the model can predict new materials, geometries, processing conditions, or equipment designs [51,87]. Therefore, the usefulness of a model should be assessed according to the level of validation evidence available. Table 6 summarizes a validation hierarchy that can be used to distinguish curve fitting, independent validation, cross-condition validation, cross-material validation, cross-equipment validation, and field-level validation.

This hierarchy shows that different validation levels support different claims. Curve fitting can be useful for estimating apparent parameters or comparing treatments, but it should not be presented as evidence of broad predictive capability [87]. Cross-condition, cross-material, and cross-equipment validation are more relevant when the goal is process optimization, scale-up, or industrial application [51]. Field-level validation is particularly important for models that claim to predict internal gradients or spatial heterogeneity. Therefore, the practical usefulness of a mass transfer model depends not only on the governing equation selected but also on the independence, diversity, and relevance of the validation evidence.

## 4. Applications of Mass Transfer Simulation in Representative Food Processing Operations

The theoretical frameworks and simulation workflow discussed in the preceding sections provide the basis for applying mass transfer modeling to practical food processes. In practical food processing systems, mass transfer does not occur in a uniform manner, but varies with the dominant transport type, structural characteristics of the material, and degree of coupling with heat transfer, fluid flow, or multicomponent interactions. Accordingly, the practical value of mass transfer simulation lies in applying governing equations and a unified simulation workflow to specific food processes so that spatial distributions can be predicted, rate-limiting steps can be identified, and quality-oriented process improvement can be supported. Therefore, this section discusses representative applications of mass transfer simulation in food processing. This work demonstrates how the governing equations and workflow elements introduced earlier are implemented in typical application scenarios. To address the mathematical implementation of these applications more explicitly, this section also summarizes the representative partial differential equations, boundary conditions, and numerical methods used in each case study, while Table 7 further evaluates their practical applicability and limitations.

### 4.1. Simulation of Drying, Dehydration, and Moisture Redistribution

**Figure 5:**
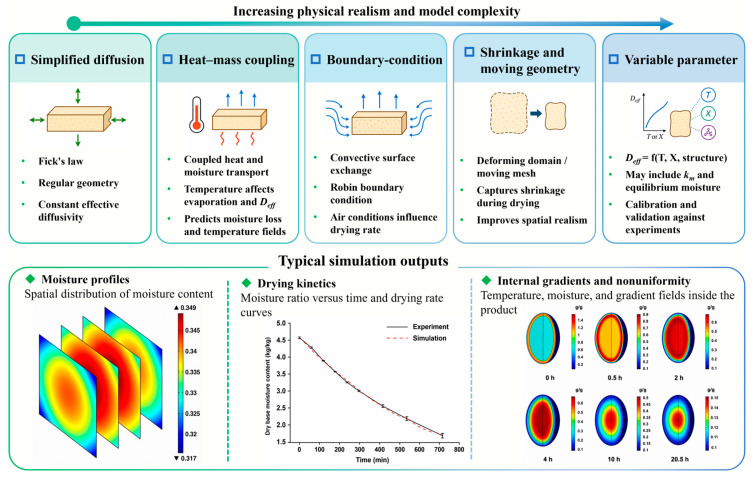
Evolution of drying and dehydration simulation from simplified Fickian diffusion to coupled models with shrinkage and variable parameters. Adapted from Refs. [100,118].

Drying and dehydration are among the most established application domains of mass transfer simulation in food processing [18,20]. In these processes, simulation is still most commonly built around Fick-based moisture transport, especially when the main objective is to estimate effective diffusivity and predict overall drying kinetics [23]. For intact biological tissues, drying simulations should also consider that blanching, pulsed electric field treatment, or mechanical disruption may modify membrane permeability and thus change the apparent effective diffusivity used in the model [57,58]. However, recent studies show a clear shift from overall diffusion fitting toward spatially resolved and structural simulation. At one level, multiscale approaches have been introduced to infer diffusivity at a small unit level during drying, suggesting that diffusion modeling can be extended beyond empirical description [51]. At another level, coupled heat and mass transfer models have been increasingly used to resolve internal temperature and moisture distributions in products such as jujube slices and shrimp during hot-air or assisted drying [70,117]. Moreover, when contraction of volume is significant, moving boundary formulations have been incorporated to better represent the evolving diffusion domain, as shown in the microwave-assisted drying of potato slices [65]. Therefore, these studies show a shift from simplified kinetic prediction to physically resolved models incorporating thermal fields, geometry evolution, and structural heterogeneity. This transition is summarized in Figure 5, which illustrates the evolution from simplified Fickian diffusion models toward heat–mass coupled transport simulations with refined boundary conditions, shrinkage-aware geometries, and variable transport parameters.

In implementation, drying and dehydration models are commonly formulated as transient moisture diffusion problems and may be extended to coupled heat–mass transfer when temperature gradients and evaporation are important [21,34]. The initial condition usually defines the initial moisture and temperature fields, whereas surface exchange is represented by convective moisture and heat boundary conditions [1,67]. Analytical or FDM-based one-dimensional solutions can be sufficient for thin slabs, plates, or sufficiently long cylinders when one transport direction dominates, and the aim is to fit drying curves or estimate effective diffusivity [34,51,74]. Under such restricted conditions, a constant effective diffusivity may be sufficient for overall kinetic prediction, provided that temperature, moisture range, shrinkage, and structural change remain limited and the model is not used to infer detailed internal gradients [52]. However, for finite solids with comparable dimensions, such as cubes, thick slices, fries, or irregular food pieces, FEM or FVM becomes more appropriate when shrinkage, irregular geometry, multidirectional gradients, or coupled heat–mass transfer need to be resolved [61]. Therefore, the practical reliability of drying simulations depends strongly on whether the selected formulation matches the intended use, from preliminary kinetic fitting to spatial prediction and process design.

### 4.2. Simulation of Frying, Baking, and Other Heat–Mass Coupled Processes

Frying, baking, and related heat–mass coupled transport operations represent a class of food processes in which mass transfer cannot be described independently of heat transfer [119,120]. Unlike diffusion-dominated drying models, these processes involve simultaneous temperature rise, moisture evaporation, vapor migration, and, in some cases, oil uptake or crust formation. For this reason, simple Fick-based descriptions may be insufficient to capture the actual transport behavior in strongly coupled thermal processes. Instead, simulation is commonly based on coupled formulations in which mass conservation equations are solved together with heat-transfer equations, while additional momentum, pressure, or deformation terms may be introduced when pore evolution, gas expansion, or structural change becomes significant [120,121]. Therefore, these processes provide a representative application domain for evaluating mass transfer simulation under strongly coupled and dynamically evolving conditions.

In practical implementation, the simulation of frying and baking usually requires careful treatment of boundary conditions, model dimensionality, and parameter variation. Surface heat and mass exchange are typically represented by convective boundary conditions, while evaporation at the food surface or within near-surface regions must be linked to local temperature and moisture conditions [120]. Depending on the model complexity, separate transport descriptions may be introduced for liquid water, water vapor, and absorbed oil, especially in frying processes where oil uptake becomes an additional response of interest. For thick or proportionally shaped products, such as potato strips, filled bakery products, or thick dough pieces, one-dimensional heat–mass transfer assumptions may underestimate multidirectional resistance and local gradients [61,121]; therefore, 2D or 3D FEM-, FVM-, or CFD-based models are more appropriate when internal temperature, moisture, vapor pressure, crust formation, or oil-uptake gradients are of interest [27,120,121].

Parameter determination is also challenging because effective diffusivity, thermal conductivity, density, and heat capacity may vary substantially with temperature, moisture content, and structural changes during processing [122]. In fried or baked products prepared from intact tissues, pretreatment-induced changes in membrane permeability may further affect moisture escape, vapor generation, crust development, and oil uptake [58,123]. In this sense, simulations of heat–mass coupled processes highlight the importance of coupled model selection, variable parameter representation, dynamic boundary treatment, and geometry-appropriate numerical implementation in food mass transfer modeling [27,124].

Mathematically, frying and baking simulations are typically implemented by coupling an energy equation with one or more species-conservation equations for moisture, vapor, or oil [24,61]. Convective heat-transfer boundaries are used to represent heat exchange with hot air, steam, or oil, while surface moisture flux, evaporation, vapor pressure, or oil-uptake conditions may be added according to the process. FEM is useful when internal heat–mass coupling, deformation, or crust formation is emphasized, whereas FVM- or CFD-based methods are more suitable when external airflow, oil flow, or equipment-scale transport controls the boundary conditions [107,119]. These models are more difficult to transfer directly to industrial systems than simple drying models because phase change, crust development, and dynamic surface conditions make the required parameters strongly process- and equipment-dependent.

### 4.3. Simulation of Curing, Osmotic Dehydration, and Solute Migration

Compared with simple diffusion and heat–mass coupled transport, explicit multicomponent transfer based on Maxwell–Stefan equations remains relatively limited in food processing applications. Nevertheless, it can be particularly useful for curing, osmotic dehydration, and solute migration, because these processes involve the concurrent redistribution of water and multiple dissolved species. Representative early applications showed that the Maxwell–Stefan framework could be used to predict simultaneous salt gain and moisture loss during cheese brining and to model coupled water and salt diffusion in dry fermented sausages, where diffusivities may depend on matrix composition, salt concentration, or water content. [42,125]. Recent work has reconsidered osmotic dehydration from a coupled multicomponent perspective, showing that uncoupled approaches cannot adequately represent the interactions among water, NaCl, and sucrose. By contrast, cross diffusion and multicomponent equilibrium provide a more rigorous description of coupled transfer in binary and ternary systems [29].

In single-component or separately fitted diffusion models, simulation is often used mainly to predict bulk water loss or solute uptake. By contrast, Maxwell–Stefan equations are more physically appropriate to situations in which competitive migration, coupled driving forces, and internal compositional redistribution are central to process behavior. This is especially relevant for curing and osmotic dehydration, where water loss, solute gain, and local concentration changes evolve simultaneously and may alter the effective transport environment during processing [29]. Although direct applications in food processing are still relatively sparse, when the research objective shifts from predicting overall mass change to analyzing the mechanistic coupling of multiple species during transfer, multicomponent transfer simulation becomes a more physically defensible choice.

In mathematical implementation, curing, osmotic dehydration, and solute migration are usually described by species-conservation equations for water and solutes. The flux term may be expressed by Fickian diffusion when each component can be treated separately, or by Maxwell–Stefan-type formulations when interactions among water, salt, sugar, or other solutes are central to the process [60,126]. Initial conditions define the original water and solute distributions inside the food, whereas boundary conditions are determined by the brine, curing solution, osmotic medium, or interfacial partition behavior [126]. FDM can be used for simple one-dimensional uptake or loss problems, but FEM is more flexible for finite, multilayer, or heterogeneous foods. The practical value of multicomponent models is highest when local concentration distribution and product uniformity are important, but it is limited when species-specific diffusivities and interfacial parameters cannot be measured or validated [64].

### 4.4. Simulation of Rehydration, Soaking, and Transport in Porous Foods

Simulation studies on rehydration, soaking, and transport in porous foods are centered on the role of internal structure in controlling liquid ingress and redistribution [127,128]. In these applications, the objective of modeling is not only to predict overall water uptake but also to describe capillary imbibition, diffusion through the food matrix, swelling, and preferential transport pathways within porous architectures. Liquid uptake during the rehydration of dry porous foods cannot be fully interpreted as bulk diffusion because capillary forces, pore connectivity, and matrix swelling strongly influence both the rate and spatial pattern of water ingress [129]. Therefore, porous-medium transport often requires structure-aware or pore-scale descriptions in which pore-space transport and solid-matrix transport are distinguished and coupled through structure-dependent exchange mechanisms [130]. Rehydration thus provides a useful case for linking food microstructure with mass transfer simulation [128].

Recent studies further indicate that the value of simulation in this category lies in linking measurable structural descriptors with predictive transport models. For example, variable diffusivity has been introduced to better represent nonuniform moisture evolution during hydration [127]. In porous vegetables and other biological tissues, pore-scale and multiscale models can be combined with MRI or CT to explain how pore connectivity, membrane disruption, blanching pretreatment, and capillary transport alter rehydration behavior. In this context, pretreatment is not only a processing variable but also a structural modifier that changes permeability, tortuosity, swelling behavior, and the accessibility of liquid pathways [54]. Recent CT-based studies relating porosity to permeability show how structural descriptors can inform predictive transport models [3]. However, simulations supported by MRI or CT should be seen mainly as research tools for structural characterization and validation, not as routine industrial methods [38]. Overall, simulation of rehydration, soaking, and porous-medium transport is moving from bulk water uptake prediction toward spatially resolved and structure-dependent analysis. In this context, porosity, permeability, tortuosity, and swelling are not only material descriptors but also physically interpretable parameters that connect food microstructure with mass transfer behavior.

From an implementation perspective, rehydration and soaking models can range from transient diffusion equations to porous-medium flow formulations. When overall water uptake is the main objective, an effective diffusion model with an initial dry or partially hydrated state and a surface water-concentration or water-activity boundary may be sufficient [127,128]. When capillary transport, pore connectivity, or pressure-driven liquid movement becomes important, Darcy-type flow can be coupled with liquid conservation, saturation, or swelling descriptions. FEM and FVM are suitable for continuum-scale porous-medium models, whereas LBM or pore-network methods are more appropriate for pore-scale transport pathways [1,130]. These detailed models are practically valuable for explaining local hydration heterogeneity and texture recovery, but their application is limited by the difficulty of measuring structure-dependent porosity, permeability, tortuosity, pore connectivity, and swelling parameters [128].

### 4.5. Simulation of Moisture Migration in Multi-Ingredient and Multilayer Foods

Multi-ingredient and multilayer foods represent an important but often less emphasized application domain of mass transfer simulation [131,132]. In products such as muesli-type mixtures, cereal–fruit systems, filled cookies, layered bakery products, and composite snacks, moisture migration is not governed only by moisture loss to the external environment. Instead, water may redistribute among components or layers with different water activity, porosity, sugar or fat content, and sorption behavior [131,133]. This internal redistribution can soften crisp components, dry or harden fillings, destabilize interfacial regions, and ultimately affect texture stability and shelf life [132,134]. Therefore, these systems provide a practical case in which mass transfer simulation is linked directly to storage stability rather than only to processing kinetics.

From an implementation perspective, multi-ingredient and multilayer foods can be represented by layer- or component-specific diffusion or conservation equations [131,132]. A representative formulation is $\partial M_{i}/\partial t=\nabla \cdot (D_{i}\nabla M_{i})$, where *M_i_* and *D_i_* denote the moisture content and effective diffusivity of the i-th component or layer. Initial conditions should define the moisture content or water activity of each component, while boundary conditions at the interface should consider the continuity of moisture flux and compatibility of water activity or sorption equilibrium [132,133]. Analytical or FDM-based solutions may be sufficient for ideal one-dimensional layered systems, whereas FEM is more suitable for finite multilayer foods, filled products, heterogeneous component arrangements, or irregular interfaces. The main practical limitation is that layer-specific diffusivity, sorption isotherms, interfacial resistance, glass-transition-related changes, and storage-dependent structural changes are difficult to determine and validate [133]. Thus, these models are most valuable when the objective is to predict shelf-life-limiting moisture redistribution rather than only average moisture equilibration.

To synthesize the representative applications discussed above and to address their mathematical implementation more explicitly, Table 7 compares the application categories in terms of representative partial differential equations or governing formulations, boundary conditions, numerical methods, practical limitations, and application relevance. Because several limitations listed in food mass transfer simulations are common phenomena rather than exceptional cases, the table further assesses the extent to which they affect practical application [51]. The impact of each limitation depends on the modeling objective: some simplifications may be acceptable for preliminary kinetic fitting, endpoint comparison, or qualitative interpretation, whereas they become problematic for spatial prediction, scale-up, shelf-life assessment, or process design [29,61,131].

**Table 7:** Mathematical implementation, practical impact, and appropriate use of representative mass transfer simulations in food processing.

Representative Processes	Representative PDEs or Governing Formulations	Typical Initial and Boundary Conditions	Mathematical or Numerical Method	Main Practical Limitation	Practical Impact on Application	Possible Mitigation or Appropriate Use	References
Drying, dehydration, and moisture redistribution	Moisture diffusion: $\frac{\partial M}{\partial t}=\nabla \cdot (D_{\mathrm{eff}}\nabla M)$ Coupled heat–mass transfer may include $\rho C_{p}\frac{\partial T}{\partial t}=\nabla \cdot (k_{\mathrm{eff}}\nabla T)-\lambda \frac{\partial M}{\partial t}$.	Initial moisture *M* = *M*_0_; initial temperature *T* = *T*_0_ if heat transfer is coupled. Surface moisture flux: $D_{\mathrm{eff}}\frac{\partial M}{\partial n}=h_{m}(M_{S}-M_{e})$. Convective heat boundary: $-k\frac{\partial T}{\partial n}=h(T_{S}-T_{\infty })$.	Analytical solutions for ideal one-dimensional slabs or cylinders; FDM for regular domains; FEM or FVM for irregular geometries, coupled heat–mass transfer, shrinkage-aware domains, or multidirectional transport.	Over-simplified geometry, constant *D*_eff_, uncertain surface transfer coefficient, shrinkage, structural evolution, and pretreatment-dependent tissue changes may reduce model reliability; inappropriate use of one-dimensional geometry for finite solids with comparable dimensions is also a major source of error.	High when the objective is to predict internal moisture gradients, drying uniformity, shrinkage, multidirectional resistance, or scale-up behavior, especially in intact tissues, thick pieces, cubes, fries, or irregular foods. Moderate to low when the objective is limited to overall drying-curve fitting or effective diffusivity estimation under narrow operating conditions.	Constant *D*_eff_ and simple geometry are acceptable for preliminary kinetic fitting under limited temperature, moisture, and shrinkage ranges. State-dependent diffusivity, shrinkage-aware geometry, 2D/3D FEM or FVM, pretreatment-specific parameters, and independent validation under different temperatures, air velocities, thicknesses, and material batches are needed for predictive or design-oriented use.	[51,52]
Frying, baking, and other heat–mass coupled processes	Energy equation: $\rho C_{p}\frac{\partial T}{\partial t}=\nabla \cdot (k\nabla T)+Q-\lambda R_{\mathrm{evap}}$ Moisture conservation: $\frac{\partial M}{\partial t}=\nabla \cdot (D_{\mathrm{eff}}\nabla M)-R_{\mathrm{evap}}$ Oil, vapor, or pressure transport may be added when needed.	Initial temperature *T* = *T*_0_; initial moisture *M* = *M*_0_; initial oil content may be zero or a measured baseline. Convective heat boundary: $-k\frac{\partial T}{\partial n}=h(T_{S}-T_{\infty })$. Surface moisture flux: $-D_{\mathrm{eff}}\frac{\partial M}{\partial n}=h_{m}(M_{S}-M_{\infty })$. Oil uptake, vapor pressure, or crust-related boundary conditions may be added in frying or baking.	FEM for coupled heat–mass and deformation problems; FVM or CFD for airflow, oil flow, or equipment-scale simulations; moving mesh may be used when deformation, crust evolution, or geometry change is important.	Strong coupling among heat transfer, evaporation, vapor migration, crust formation, oil uptake, tissue structure, and dynamic boundary conditions makes model parameterization difficult. One-dimensional heat–mass assumptions may be insufficient for thick or proportionally shaped products.	Very high for quantitative prediction of oil uptake, crust formation, internal temperature, moisture redistribution, and product quality. These limitations strongly restrict direct transfer from laboratory models to industrial fryers or ovens. Moderate when the model is used only for endpoint comparison or qualitative mechanism interpretation.	Reduced models may be sufficient for endpoint comparison. Coupled heat–mass formulations, temperature-dependent properties, dynamic boundary conditions, 2D/3D numerical domains, and validation under different oil/air temperatures, product dimensions, and equipment configurations are required for process design or scale-up. Tissue state and pretreatment history should be reported when fitted water/oil transfer parameters are used.	[24,61]
Curing, osmotic dehydration, and solute migration	Species conservation: $\frac{\partial C_{i}}{\partial t}=-\nabla \cdot N_{i}$; Fickian flux: $J_{i}=-D_{i}\nabla C_{i}$; Maxwell–Stefan formulations can be used when water–solute or solute–solute interactions are important.	Initial concentration *C_i_* = *C_i_*_,0_ in the food matrix; surface concentration, partition, or interfacial mass transfer boundary defined by brine, curing solution, or osmotic medium.	FDM for simple one-dimensional diffusion; FEM for finite, multilayer, or heterogeneous geometries; numerical solvers are required for coupled Maxwell–Stefan systems.	Species diffusivities, interaction parameters, partition coefficients, interfacial mass transfer resistance, and component-specific concentration profiles are difficult to determine and validate.	High when the goal is to predict local salt, sugar, or water distributions, product uniformity, or safety-related concentration gradients. Moderate to low when only total water loss or total solid gain is required.	Fickian or lumped approaches are acceptable for bulk uptake or loss prediction. Maxwell–Stefan or multicomponent models are more appropriate when competitive diffusion, cross-effects, and internal composition profiles are central. Both bulk mass changes and local concentration profiles should be validated when product uniformity is claimed.	[29,42]
Rehydration, soaking, and transport in porous foods	Diffusion-based uptake: $\frac{\partial M}{\partial t}=\nabla \cdot (D_{\mathrm{eff}}\nabla M)$. Darcy-type flow: u=−(K/μ)∇p. Liquid conservation may be coupled with saturation, swelling, or capillary transport equations.	Initial dry or partially hydrated state *M* = *M*_0_; initial saturation or pressure field if porous-medium flow is modeled. Surface water concentration, water activity, saturation, or pressure boundary; no-flux boundary for impermeable surfaces; moving boundary may be used when swelling is significant.	FEM or FVM for continuum porous-medium models; LBM or pore-network methods for pore-scale transport; image-based meshes when pore geometry is reconstructed from CT, MRI, or other imaging methods.	Porosity, permeability, tortuosity, pore connectivity, swelling, membrane disruption, and pretreatment-induced structural changes are strongly material-dependent and difficult to parameterize. Pore-scale validation is also difficult.	High when predicting liquid penetration pathways, local hydration heterogeneity, swelling, or texture recovery. Moderate when only the total water uptake or the empirical rehydration ratio is needed.	Continuum diffusion models are suitable for overall water uptake. Porous-medium, pore-network, LBM, or image-based models are needed when structure-dependent pathways, capillary transport, swelling, or pore connectivity determine process performance. Parameters should be validated across different porosities, pretreatments, maturity, and drying histories.	[35,36]
Multi-ingredient and multilayer foods	Diffusion/conservation equations specific to layers or components, $\frac{\partial M_{i}}{\partial t}=\nabla \cdot (D_{i}\nabla M_{i})$ where *M_i_* and *D_i_* denote the moisture content and effective diffusivity of the *i*-th component or layer.	Initial moisture or water activity differs among components. Interfacial conditions require continuity of moisture flux and compatibility of water activity or sorption equilibrium. External packaging or storage humidity may define outer boundary conditions.	Analytical or FDM-based one-dimensional models are suitable for planar-layered systems when moisture transfer is mainly perpendicular to the layers. FEM is more appropriate for finite filled products, irregular interfaces, heterogeneous component arrangements, or multidirectional moisture redistribution.	Component-specific diffusivities, sorption isotherms, interfacial resistance, glass transition behavior, and storage-dependent structural changes are difficult to determine and validate.	High for shelf-life prediction when moisture redistribution causes crispness loss, filling hardening, microbial risk, or interfacial instability. Moderate when only average moisture equilibration is required.	Use layer-specific parameters and sorption isotherms. Validate moisture profiles and water activity changes during storage, especially at interfaces. Use simplified 1D models for planar-layered products and FEM for finite or irregular composite products.	[131,132]

As shown in Table 7, the limitations of mass transfer simulation should not be interpreted uniformly. Their practical significance depends on whether the model is used for curve fitting, mechanism interpretation, spatial prediction, process design, scale-up, or shelf-life assessment. Constant diffusivity and one-dimensional geometry may be acceptable for narrow-range drying-curve fitting, but they become limiting when internal gradients, shrinkage, multidirectional resistance, or scale-up behavior need to be predicted. Similarly, simplified multicomponent, porous-medium, or multilayer models may be sufficient for bulk uptake or average moisture equilibration but not for local concentration profiles, texture recovery, or interfacial moisture redistribution [132]. Therefore, practical applicability should be assessed according to the intended use of the model and the validation evidence available rather than by listing model limitations alone [69].

## 5. Challenges and Future Perspectives

Several limitations still constrain the predictive use of mass transfer modeling and simulation in food processing. A major limitation is the mismatch between simplified theoretical formulations and the structural complexity of real food materials. Although Fick-based diffusion equations, Darcy-based porous-medium formulations, and Maxwell–Stefan or other multicomponent equations provide useful starting points, their predictive performance often deteriorates when tissue heterogeneity, cell membrane resistance, structural evolution, and coupled transport effects become significant [22]. Inappropriate dimensional reduction, especially the use of one-dimensional diffusion equations for finite solids with comparable dimensions, can lead to underestimation of multidirectional heat and mass resistance [37]. Future models should better link governing equations with evolving food geometry, properties, and structure [21,135].

Model transferability is also limited by the biological state of the food material. In intact tissues, effective diffusivity and permeability may partly reflect cell membrane resistance, tissue connectivity, and pretreatment-induced structural changes [59]. Blanching, pulsed electric field treatment, freezing, cutting, or mechanical disruption can alter membrane permeability and transport pathways, meaning that parameters fitted for untreated samples may not be transferable to pretreated or powdered materials [57]. Future studies should therefore report the tissue state and pretreatment history more explicitly and validate the transport parameters across biologically different material states.

Another challenge concerns the rigor and transparency of the simulation workflow. In many studies, geometric simplification, constant parameter assumptions, and idealized boundary conditions remain necessary, but they also restrict model reliability and transferability. These simplifications are most defensible when the model is used for narrow-range kinetic fitting, but they become problematic when the goal is to predict spatial fields, cross-condition behavior, or process scale-ups [93,122]. Workflow rigor requires clearer links among image-informed geometry, variable parameters, uncertainty analysis, and physically justified initial and boundary conditions [38,122].

Validation remains another major bottleneck. Bulk measurements are still widely used, but they are often insufficient for assessing spatially resolved predictions in heterogeneous, multicomponent, or porous food systems. More robust validation requires closer integration of simulations with imaging and spatial characterization methods so that internal gradients, concentration fields, and structural changes can be evaluated more directly. Recent studies combining simulation with quantitative magnetic resonance imaging (MRI) and multi-output baking validation illustrate the value of this direction [14,124]. A related issue is model transferability. Many food mass transfer models are inverse-calibrated using data obtained from specific material, geometry, equipment designs, and boundary conditions [52]. As a result, good agreement with the calibration data does not guarantee that the same model will remain valid for different varieties, maturity stages, pretreatments, sample dimensions, airflow or oil flow patterns, or equipment configurations [122]. Future studies should therefore distinguish parameter fitting from independent validation and should report the range of materials, boundary conditions, and equipment settings over which a model has been tested.

Limited industrial adoption is another important challenge. Although advanced simulations can provide spatially resolved information on moisture, temperature, solute, oil, or gas transport, their direct use in routine production remains limited because accurate simulations require product-specific geometry, reliable material properties, well-defined boundary conditions, validation data, specialized software, computational resources, and expert operation [107]. Advanced simulations based on MRI, CT, or other expensive imaging data are valuable for mechanism interpretation, model construction, and validation, but they are rarely feasible as routine industrial tools [38]. At present, the more realistic industrial value of advanced modeling lies in offline equipment design, airflow or heat-transfer analysis, scale-up support, process troubleshooting, and pre-production optimization.

Overall, future progress in this field should not be framed simply as increasing model complexity but as improving the connections among governing equations, workflow transparency, parameter determination, and application validation. Emerging but still preliminary directions include artificial-intelligence-assisted modeling, physics-informed or hybrid surrogate strategies for field prediction and parameter estimation [72], and digital twins for data–simulation linkage [136]. These approaches may help learn state-dependent parameters, correct model bias, and connect accessible process measurements with simplified mechanistic models but their practical value still depends on physical interpretability, independent validation, and transferability across materials and equipment [72,137].

## 6. Conclusions

Food mass transfer is better understood by connecting process descriptions with governing equations, workflow design, and representative applications. The literature indicates that no single transport framework is universally applicable. Appropriate model selection depends on the dominant transport type, the degree of coupling involved, and the structural complexity of the food matrix. At the same time, predictive reliability is determined not only by equation choice but also by geometric representation, parameter determination, boundary condition specification, numerical implementation, independent validation, and transferability assessment. Across drying, heat–mass coupled transport, multicomponent transfer, porous-medium transport, and moisture redistribution in composite foods, recent studies increasingly use spatially resolved, structure-aware, and mechanism-oriented simulations. The biological state of food tissues, including cell membrane integrity and pretreatment-induced structural changes, should be considered when interpreting fitted transport parameters and assessing model transferability. Future frameworks should better connect equation selection, parameter representation, boundary specification, numerical implementation, validation, industrial applicability assessment, and model simplification. Artificial intelligence may further contribute to application-oriented models by assisting parameter estimation, surrogate prediction, bias correction, and the integration of accessible process measurements with physically based transport models, provided that these models are physically interpretable and independently validated.

## Figures and Tables

**Table 1:** Fundamental transport laws, representative equations, and geometric applicability in food processing simulations.

Transport Process	Governing Law or Formulation	Representative Equation	Definition of Terms	Steady/Unsteady Use	Geometry/Application Criterion	References
Momentum transfer	Newton’s law of viscosity	$\tau =\mu \frac{du}{dy}$	τ: shear stress; *μ*: dynamic viscosity; u: velocity; y: coordinate normal to flow direction	Describes local viscous momentum transfer under steady or transient flow conditions; often used as a constitutive relation in flow models	Relevant to external airflow, oil flow, vapor movement, boundary layers, pores, or channels; geometry is defined by the fluid domain or pore structure rather than by the solid food shape alone	[1,28]
Heat conduction	Fourier’s law	$q^{n}=-k\nabla T$	q^n^: conductive heat flux; *k*: thermal conductivity; T: temperature	Steady conduction can be described when the temperature does not change with time; unsteady conduction is used when T evolves during processing	Applicable to slabs, cylinders, spheres, finite solids, or irregular geometries; multidimensional models are required when temperature gradients exist in several directions	[21,27]
Convective heat transfer	Newton’s law of cooling	$q^{n}=h(T_{\infty }-T_{s})$	h: convective heat-transfer coefficient; $T_{\infty }$: surrounding medium temperature; *T_s_*: surface temperature	Usually used as a boundary condition for steady or unsteady heat-transfer problems	Applies to exposed food surfaces in contact with hot air, steam, oil, or cooling medium; depends on surface geometry, flow regime, and characteristic length	[24,26]
Steady mass diffusion	Fick’s first law	J=−D∇C	*J*: diffusion flux; *D*: diffusion coefficient; *C*: concentration of the diffusing component	Used for steady or quasi-steady diffusion flux driven by a concentration gradient	Suitable when a dominant diffusion direction can be defined; common in thin slabs or simplified one-dimensional diffusion paths	[22,23]
Transient mass diffusion	Fick’s second law	$\frac{\partial C}{\partial t}=\nabla \cdot (D\nabla C)$	*C*: concentration; *t*: time; *D*: diffusion coefficient	Used for unsteady diffusion when concentration changes with time	One-dimensional analytical forms are appropriate only when one transport direction dominates, such as infinite slabs/plates or infinite cylinders. Finite solids with comparable dimensions require 2D or 3D formulations because heat and mass resistance may be significant in multiple directions	[21,23]
General species conservation	Conservation-based mass balance	$\frac{\partial C_{A}}{\partial t}+\nabla \cdot N_{A}=R_{A}$	*C_A_*: concentration of component A; *N_A_*: total flux of component A; *R_A_*: source or sink term of component A	Used mainly for unsteady transport with convection, diffusion, source terms, phase change, or reaction	Suitable for coupled heat–mass transfer, vapor migration, oil uptake, phase transition, or interfacial exchange; geometry depends on the simulated food domain	[1,24]
Pressure-driven flow in porous foods	Darcy’s law	$q=-\frac{k}{\mu }\nabla p$	q: Darcy velocity; k: permeability; μ: dynamic viscosity; p: pressure	Can be used under steady or transient pressure-driven flow when inertial effects are limited	Applicable to porous foods such as bread, cakes, dried fruits, grains, and rehydrated matrices; requires a representative porous-medium description	[1,27]
Multicomponent diffusion	Maxwell–Stefan formulation	$-\nabla x_{i}=\sum_{j\neq i}\frac{x_{j}N_{i}-x_{i}N_{j}}{cD_{ij}}$	$x_{i}$: mole fraction of component *i*, $N_{i}$: molar flux, c: total molar concentration; $D_{ij}$: Maxwell–Stefan diffusivity	Used for steady or unsteady multicomponent systems when species interactions are important	Relevant to osmotic dehydration, curing, marination, salting, and sugar/salt/water redistribution; usually requires a numerical solution in finite or irregular geometries	[22,29]

**Table 2:** Dimensionless numbers related to momentum, heat, and mass transfer in food processing simulations.

Dimensionless Number	Symbol	Equation	Related Transfer Process	Physical Meaning in Food Processing	References
Reynolds number	Re	$Re=\frac{\rho UL_{c}}{\mu }$	Momentum transfer	Ratio of inertial to viscous forces; indicates flow regime around or through food materials	[26]
Prandtl number	Pr	$Pr=\frac{\mu C_{p}}{k}=\frac{v}{\alpha }$	Momentum–heat transfer	Ratio of momentum diffusivity to thermal diffusivity; relates the velocity boundary layer to the thermal boundary layer	[26]
Schmidt number	Sc	$Sc=\frac{\mu }{\rho D}=\frac{\nu }{D}$	Momentum–mass transfer	Ratio of momentum diffusivity to mass diffusivity; relates the velocity boundary layer to the concentration boundary layer	[22]
Nusselt number	Nu	$Nu=\frac{hL_{c}}{k_{f}}$	Heat transfer	Ratio of convective to conductive heat transfer; used to estimate the external heat-transfer coefficient	[24,26]
Sherwood number	Sh	$Sh=\frac{h_{m}L_{c}}{D}$	Mass transfer	Ratio of convective to diffusive mass transfer; used to estimate the external mass-transfer coefficient	[22,24]
Heat Biot number	$Bi_{h}$	$Bi_{h}=\frac{hL_{c}}{k_{s}}$	Heat transfer	Ratio of internal conductive resistance to external convective resistance; helps judge whether internal temperature gradients are important	[21,24]
Mass Biot number	$Bi_{m}$	$Bi_{m}=\frac{h_{m}L_{c}}{D}$	Mass transfer	Ratio of internal diffusive resistance to external mass transfer resistance; helps evaluate surface resistance versus internal diffusion control	[22,23]
Heat Fourier number	$Fo_{h}$	$Fo_{h}=\frac{\alpha t}{L_{c}^{2}}$	Unsteady heat transfer	Dimensionless time for heat conduction; indicates the progress of transient temperature equalization	[24,27]
Mass Fourier number	$Fo_{m}$	$Fo_{m}=\frac{Dt}{L_{c}^{2}}$	Unsteady mass transfer	Dimensionless time for diffusion; indicates the progress of transient moisture or solute redistribution	[22,23]
Peclet number	Pe	$Pe=\frac{uL_{c}}{D}$	Convection–diffusion mass transfer	Ratio of convective to diffusive transport; important when flow contributes to internal or external mass transfer	[1,29]
Lewis number	Le	$Le=\frac{\alpha }{D}$	Coupled heat–mass transfer	Ratio of thermal diffusivity to mass diffusivity; indicates whether heat and mass transfer occur at comparable rates	[1,21]

*ρ*: density; U: characteristic velocity; *Lc*: characteristic length; *μ*: dynamic viscosity; *C_p_*: specific heat capacity; *k_f_*: thermal conductivity of fluid or medium; k_S_: thermal conductivity of solid food; *D*: diffusion coefficient; *ν*: kinematic viscosity; α: thermal diffusivity; *h*: cnvective heat-transfer coefficient; *h*_m_: convective mass-transfer coefficient; *t*: time.

**Table 3:** Comparative summary of governing equation frameworks for food mass transfer simulation.

Governing Equation Framework	Transport Conditions	Main Assumptions	Representative Food Processing Applications	Main Advantages	Main Limitations or Cautions	References
Fick’s first law	Steady or quasi-steady diffusion driven by a concentration or moisture gradient	Diffusion is the dominant mechanism; flux is proportional to the concentration gradient; material properties are often treated as constant or apparent	Steady moisture or solute diffusion; preliminary interpretation of diffusion flux; simplified drying, soaking, curing, or rehydration analysis	Simple physical meaning; few parameters; useful for estimating flux and interpreting gradient-driven transport	Not suitable for strongly transient, coupled, multicomponent, pressure-driven, or structurally evolving systems unless used as a local or simplified approximation	[22,51]
Fick’s second law	Unsteady diffusion with time-dependent concentration or moisture fields	Diffusion dominates; convection, pressure-driven flow, phase change, and strong multicomponent interactions are negligible or incorporated into an effective diffusivity	Drying curves, moisture redistribution, soaking, rehydration, curing, and dehydration of simple geometries	Widely used; supports analytical and numerical solutions; useful for estimating effective diffusivity and predicting overall kinetics	One-dimensional analytical forms are valid mainly for thin slabs, plates, or long cylinders. Finite solids with comparable dimensions require 2D or 3D formulations. Constant *D*_eff_ is acceptable mainly under narrow temperature/moisture ranges and limited structural change	[34,52]
General species conservation equation	Diffusion coupled with convection, phase change, source/sink terms, interfacial exchange, or reaction	Mass balance is written for each transported component; total flux may include diffusive and convective contributions; source terms are defined according to the process	Coupled drying, frying, baking, vapor migration, oil uptake, evaporation, condensation, and reaction-related transport	More flexible than pure Fickian diffusion; can incorporate flow, generation/consumption terms, and boundary exchange	Requires more parameters, boundary conditions, and validation data; poorly defined source terms or flux expressions may reduce physical interpretability	[1,21]
Coupled heat–mass transfer equations	Moisture or solute transport is strongly coupled with temperature evolution, evaporation, or phase change	Heat transfer affects diffusivity, vapor pressure, evaporation rate, and boundary flux; mass transfer may feed back through latent heat or structural change	Hot-air drying, microwave drying, baking, frying, roasting, and other thermal processes	Captures interaction between temperature fields and moisture redistribution; useful for predicting internal gradients and non-uniformity	Requires thermal properties, latent heat terms, heat-transfer coefficients, and moisture-dependent parameters; model transferability is limited if boundary conditions are equipment-specific	[1,24]
Maxwell–Stefan equations	Multicomponent diffusion with strong species interactions or coupled driving forces	Frictional interactions among species are considered; the flux of one species depends on the movement and concentration of others	Osmotic dehydration, curing, salting, marination, brining, sugar/salt/water redistribution	More physically rigorous than independent Fickian diffusion for coupled multicomponent systems	Requires interaction diffusivities and concentration-dependent parameters; a numerical solution is often needed; validation of local concentration profiles is difficult	[22,29]
Darcy’s law	Pressure-driven liquid or gas flow through porous foods	Flow occurs through a representative porous medium; permeability and viscosity govern pressure-driven transport; inertial effects are limited	Rehydration, soaking, porous drying, liquid penetration, and gas movement in porous bakery or dried foods	Provides a simple framework for linking pressure gradients, permeability, and flow in porous structures	Permeability is material- and structure-dependent; pore connectivity, swelling, shrinkage, and multiphase effects may require additional equations	[1,51]
Porous-medium or multiphase transport models	Coupled liquid, vapor, gas, or capillary transport in porous matrices	Phases are represented by saturation, pressure, permeability, capillary pressure, or phase-change terms	Drying of porous foods, grains, bakery products, rehydration of dried porous materials, and capillary liquid ingress	Can represent liquid/vapor movement, pore resistance, capillary effects, and structure-dependent pathways	Parameterization is difficult; porosity, tortuosity, permeability, saturation, and capillary parameters may change during processing	[1,51]
Empirical, inverse, or hybrid parameterized models	Mechanistic equations combined with fitted parameters, correction functions, or data-driven components	Model structure is partly physics-based, but key parameters are fitted from experimental data	Drying kinetics, hydration curves, salting/osmotic uptake, process comparison, surrogate modeling	Useful when direct parameter measurement is difficult; can improve fitting and practical prediction within calibrated ranges	Fitting should not be confused with independent validation; transferability to new materials, geometries, equipment, or boundary conditions requires additional testing	[34,52]

**Table 4:** Recent advances in governing formulations and modeling strategies for food drying simulations.

Modeling Advance	Representative Formulation or Equation	Main Improvement over Classical Fickian Models	Target Drying Issue or Application Scenario	Workflow Implication	References
State-dependent effective diffusivity	$\frac{\partial M}{\partial t}=\nabla \cdot [D_{\mathrm{eff}}(T,M)\nabla M]$	Replaces a constant effective diffusivity with a function of temperature, moisture content, or local material state	Nonlinear drying kinetics caused by changing moisture content and temperature	Requires parameter functions, sensitivity analysis, and validation under multiple drying conditions	[51,52]
Coupled heat–mass transfer	$\begin{array}{l} \rho C_{p}\frac{\partial T}{\partial t}=\nabla \cdot (k_{s}\nabla T)-\lambda \frac{\partial M}{\partial t};\\ \frac{\partial M}{\partial t}=\nabla \cdot (D_{\mathrm{eff}}\nabla M) \end{array}$	Links temperature evolution, evaporation, and moisture migration instead of treating moisture diffusion alone	Drying processes where temperature gradients and evaporation strongly affect moisture movement	Requires simultaneous solution of heat and mass equations and consistent thermal and moisture boundary conditions	[1,21]
Shrinkage- or deformation-coupled drying model	$\frac{\partial M}{\partial t}=\nabla \cdot (D_{\mathrm{eff}}\nabla M)$ in a moving domain Ω(*t*); *V*/*V*_0_ = *f* (*M*)	Updates the computational domain as the food shrinks or deforms during moisture loss	Fruit, vegetables, potatoes, and gel drying, with significant volume or shape change	Requires geometry updating, moving mesh, adaptive mesh, or empirical shrinkage functions	[1,65]
Porous-medium and multiphase transport	$\begin{array}{l} \frac{\partial (\varepsilon S_{i}\rho_{i})}{\partial t}+\nabla \cdot (\rho_{i}u_{i})=R_{i};\\ u_{i}=-\frac{Kk_{ri}}{\mu_{i}}\nabla p_{i} \end{array}$	Represents liquid/vapor movement, capillary effects, pressure gradients, and pore resistance	Drying of porous foods, grains, bakery products, and high-porosity matrices and pressure gradients are important	Requires porosity, permeability, saturation, pressure, and phase-change parameters	[1,66]
CFD-coupled drying model	Navier–Stokes equations + energy equation + species transport equation, coupled with surface heat and moisture fluxes	Couples external airflow with product surface heat and mass transfer	Convective drying is affected by airflow distribution, turbulence, tray position, or dryer design	Requires an external fluid domain, turbulence model, surface-flux boundary, and mesh-independence test	[26,67]
Electromagnetic-assisted drying model	$\rho C_{p}\frac{\partial T}{\partial t}=\nabla \cdot (k\nabla T)+Q_{EM}-\lambda \frac{\partial M}{\partial t}$	Adds volumetric heat generation from microwave or radiofrequency energy	Microwave, radiofrequency, or hybrid drying with nonuniform internal heating	Requires an electromagnetic power-absorption term and coupling with heat–mass transfer	[65,68]
Image-based or structure-informed geometry	Classical transport equations solved in domains derived from CT, MRI, HSI, or 3D scans	Improves representation of irregular shape, tissue heterogeneity, pores, or anisotropic pathways	Drying of irregular, porous, or structurally heterogeneous foods	Requires image segmentation, geometry reconstruction, mesh generation, and field-level validation	[69,70]
Pore-scale or lattice-based transport	LBM, pore-network, or microstructure-resolved diffusion/convection formulations	Resolves local transport pathways rather than treating the food as a homogeneous continuum	Drying in highly porous or heterogeneous microstructures	Requires pore-scale geometry, high computational cost, and multiscale interpretation	[71,72]
Hybrid or inverse-parameterized models	Mechanistic PDE + fitted *D*_eff_, h_m_, h, or correction functions.	Improves fitting and prediction when direct parameter measurement is difficult	Drying systems with unknown or state-dependent transport parameters	Requires independent validation to avoid overfitting and to test transferability	[52,73]

Note: M, moisture content; T, temperature; *D*_eff_, effective diffusivity; ρ, density; *C_p_*, specific heat capacity; k, thermal conductivity; λ, latent heat term; Ω(t), time-dependent computational domain; V/V0, shrinkage ratio; ε, porosity; S_i_, saturation of phase i; ρi, density of phase i;*ν_i_*, phase velocity of phase *i*; R_i_, source/sink term of phase or component *i*; K, permeability; k_ri_, relative permeability; *μ_i_*, dynamic viscosity; *p_i_*, pressure; *Q*_EM_, electromagnetic heat-generation term; CT, computed tomography; MRI, magnetic resonance imaging; HSI, hyperspectral imaging; LBM, lattice Boltzmann method; PDE, partial differential equation.

**Table 5:** Common simulation tools, model types/physical modules, and representative applications in food mass transfer studies.

Simulation Tools	Model Types/Physical Modules	Representative Applications	References
COMSOL Multiphysics	Transport of diluted species; Darcy or porous-medium flow; heat transfer; custom PDE modules for Maxwell–Stefan and osmotic transport	Potato drying; freezing of vacuum-packed beef	[98,100]
ANSYS Fluent	CFD-based momentum, heat, and species transport; turbulence; multiphase flow; conjugate heat transfer	Milk concentration; carrot drying	[101,102]
OpenFOAM	CFD-based heat–mass transfer; multiphase and porous-medium flow; conjugate transport	Drying of pineapple with hot air; spray drying	[99,103]
MATLAB	Fickian diffusion; parameter inversion and diffusivity fitting; custom PDE/ODE solvers	Estimation of water diffusivity in milk powder; goat meat drying kinetics	[104,105]

**Table 6:** Validation levels and transferability assessment for food mass transfer models.

Validation Level	Validation Evidence	What It Can Support	Main Limitation	Transferability Implication	References
Curve fitting to calibration data	Model output is compared with the same data used for parameter estimation, such as drying curves, water uptake curves, or solute gain/loss curves	Descriptive agreement under one specific material and process condition	Does not prove prediction ability because parameters may be overfitted	Low transferability; valid mainly for the calibrated condition	[34,87]
Independent validation under the same conditions	Parameters are estimated from one dataset and tested using independent replicates under the same material and operating conditions	Reproducibility of the model under controlled conditions	Still limited to the same material, geometry, and equipment setup	Moderate transferability within the same process window	[34,51]
Cross-condition validation	The model is tested under different temperatures, air velocities, oil temperatures, humidity levels, solution concentrations, and process durations	Predictive ability across operating conditions	Requires state-dependent parameters or a robust boundary condition description	Higher transferability within the same material and equipment type	[34,52]
Cross-material validation	The model is tested on materials with different varieties, maturity, composition, tissue integrity, pretreatment, or structure	Robustness to biological and structural variability	Difficult because diffusivity, permeability, and sorption properties may change substantially	Essential before generalizing the model to different foods or raw material batches	[20,51]
Cross-geometry or cross-scale validation	The model is tested on different sample sizes, shapes, thicknesses, and scale-up conditions	Ability to represent multidirectional transport and scale effects	One-dimensional assumptions or fitted coefficients may fail when geometry changes	Important for process design and industrial scaling	[107,115]
Cross-equipment validation	The model is tested in different dryers, fryers, ovens, soaking systems, or airflow/oil flow configurations	Practical usefulness beyond a single apparatus	Boundary conditions and transfer coefficients may be equipment-specific	Required for industrial application or equipment design	[26,107]
Field-level or spatial validation	Predicted moisture, temperature, solute, or oil distributions are compared with imaging, spatial sampling, MRI, CT, HSI, or other field-resolved measurements	Reliability of internal gradients and nonuniform distribution predictions	More expensive and technically demanding than bulk validation	Strong evidence for structure-aware and mechanism-oriented models	[69,116]

## Data Availability

No new data were created or analyzed in this study. Data sharing is not applicable to this article.

## References

[1] Zhu Y., Wang P., Sun D., Qu Z., Yu B. (2021). Multiphase porous media model with thermo-hydro and mechanical bidirectional coupling for food convective drying. Int. J. Heat Mass Transf..

[2] Zewdie T.A., Delele M.A., Fanta S.W., Alemayehu M., Alemayehu G., Adgo E., Nyssen J., Verboven P., Nicolai B.M. (2022). Optimisation of onion bulb curing using a heat and mass transfer model. Biosyst. Eng..

[3] Gao H., Zhu Z., Sun D.-W. (2025). Determination of porosity and permeability correlation of leafy vegetable based on X-ray computed tomography and cell segmentation. J. Food Eng..

[4] Dai B., Kan A., Li F., Gao J., Yi B., Cao D. (2022). A cross-regional thermo-hydro transport model for vacuum pre-cooling. J. Food Eng..

[5] Huang Z., Kan A., Lu J., Li F., Wang T. (2021). Numerical simulation and experimental study of heat and mass transfer in cylinder-like vegetables during vacuum cooling. Innov. Food Sci. Emerg. Technol..

[6] Lee S.H., Choi W., Jun S. (2016). Conventional and Emerging Combination Technologies for Food Processing. Food Eng. Rev..

[7] Knorr D., Augustin M.A. (2021). Food processing needs, advantages and misconceptions. Trends Food Sci. Technol..

[8] Dadmohammadi Y., Datta A.K. (2022). Food as porous media: A review of the dynamics of porous properties during processing. Food Rev. Int..

[9] Datta A., Nicolaï B., Vitrac O., Verboven P., Erdogdu F., Marra F., Sarghini F., Koh C. (2022). Computer-aided food engineering. Nat. Food.

[10] Li J., Shi J., Wang T., Huang X., Zou X., Li Z., Zhang D., Zhang W., Xu Y. (2021). Effects of pulsed electric field pretreatment on mass transfer kinetics of pickled lotus root (*Nelumbo nucifera* Gaertn.). LWT.

[11] Ni J.-B., Zielinska M., Wang J., Fang X.-M., Prakash Sutar P., Li S.-B., Li X.-X., Wang H., Xiao H.-W. (2023). Post-harvest ripening affects drying behavior, antioxidant capacity and flavor release of peach via alteration of cell wall polysaccharides content and nanostructures, water distribution and status. Food Res. Int..

[12] Fadiji T., Ashtiani S.H.M., Onwude D.I., Li Z., Opara U.L. (2021). Finite Element Method for Freezing and Thawing Industrial Food Processes. Foods.

[13] Chigwaya K., Plessis A.D., Viljoen D.W., Crouch I.J., Crouch E.M. (2021). Use of X-ray computed tomography and 3D image analysis to characterize internal browning in ‘Fuji’ apples after exposure to CO_2_ stress. Sci. Hortic..

[14] Monod R., Clerjon S., Sicard J., Pagés G., Bonny J.-M. (2025). Spatiotemporal quantification of sodium concentration in food using magnetic resonance imaging. Food Res. Int..

[15] Shi Y., Wang Y., Hu X., Li Z., Huang X., Liang J., Zhang X., Zhang D., Zou X., Shi J. (2023). Quantitative characterization of the diffusion behavior of sucrose in marinated beef by HSI and FEA. Meat Sci..

[16] Szpicer A., Bińkowska W., Stelmasiak A., Zalewska M., Wojtasik-Kalinowska I., Piwowarski K., Piepiórka-Stepuk J., Półtorak A. (2025). Computational Fluid Dynamics Simulation of Thermal Processes in Food Technology and Their Applications in the Food Industry. Appl. Sci..

[17] Akter F., Muhury R., Sultana A., Deb U.K. (2022). A Comprehensive Review of Mathematical Modeling for Drying Processes of Fruits and Vegetables. Int. J. Food Sci..

[18] Baidhe E., Clementson C.L. (2024). A review of the application of modeling and simulation to drying systems for improved grain and seed quality. Comput. Electron. Agric..

[19] Dehghannya J., Ngadi M. (2021). Recent advances in microstructure characterization of fried foods: Different frying techniques and process modeling. Trends Food Sci. Technol..

[20] Wijerathne A.D.H.T., Joardder M.U.H., Welsh Z.G., Nayak R., Sablani S.S., Karim A. (2025). Recent Advances in Food Drying Modeling: Empirical to Multiscale Physics-Informed Neural Networks. Compr. Rev. Food Sci. Food Saf..

[21] Li J., Deng Y., Xu W., Zhao R., Chen T., Wang M., Xu E., Zhou J., Wang W., Liu D. (2023). Multiscale modeling of food thermal processing for insight, comprehension, and utilization of heat and mass transfer: A state-of-the-art review. Trends Food Sci. Technol..

[22] González-Pérez J.E., Ramírez-Corona N., López-Malo A. (2021). Mass Transfer During Osmotic Dehydration of Fruits and Vegetables: Process Factors and Non-Thermal Methods. Food Eng. Rev..

[23] Welsh Z.G., Khan M.I.H., Karim M.A. (2021). Multiscale modeling for food drying: A homogenized diffusion approach. J. Food Eng..

[24] Dash K.K., Sharma M., Tiwari A. (2022). Heat and mass transfer modeling and quality changes during deep fat frying: A comprehensive review. J. Food Process Eng..

[25] Rana A., Dhiman A., Kumar S., Suhag R., Saini R. (2024). Osmosonication for dehydration of fruits and vegetables: Mechanistic understanding, mathematical models and comprehensive applications in processing. Trends Food Sci. Technol..

[26] Szpicer A., Bińkowska W., Wojtasik-Kalinowska I., Salih S.M., Półtorak A. (2023). Application of computational fluid dynamics simulations in food industry. Eur. Food Res. Technol..

[27] Ghaitaranpour A., Koocheki A., Mohebbi M. (2024). Simulation of bread baking with a conceptual agent-based model: An approach to study the effect of proofing time on baking behavior. J. Food Eng..

[28] Al-Najjar S.Z., Al-Sharify Z.T., Onyeaka H., Miri T., Obileke K., Anumudu C.K. (2023). Advances in mass transfer and fluid flows in non-thermal food processing industry—A review. Food Prod. Process. Nutr..

[29] Ramos-Morales M., Estévez-Sánchez K.H., Corona-Jiménez E., Sánchez-Cantú M., Cortés-Zavaleta O., Ochoa-Velasco C.E., Ruiz-López I.I. (2026). Exploring multicomponent equilibrium and cross-diffusion in osmotic dehydration: A new perspective on mass transfer. J. Food Eng..

[30] Khan M.I.H., Batuwatta-Gamage C.P., Karim M.A., Gu Y. (2022). Fundamental Understanding of Heat and Mass Transfer Processes for Physics-Informed Machine Learning-Based Drying Modelling. Energies.

[31] Nguyen T.T., Rosselló C., Ratti C. (2023). Simple mathematical modelling to represent air-drying kinetics of potato peel. J. Food Eng..

[32] Zhou L., Nyberg K., Rowat A.C. (2015). Understanding diffusion theory and Fick’s law through food and cooking. Adv. Physiol. Educ..

[33] Vahidhosseini S.M., Barati E., Esfahani J.A. (2016). Green’s function method (GFM) and mathematical solution for coupled equations of transport problem during convective drying. J. Food Eng..

[34] Vega-Castro O., Osorio-Arias J., Duarte-Correa Y., Jaques A., Ramírez C., Núñez H., Simpson R. (2023). Critical Analysis of the Use of Semiempirical Models on the Dehydration of Thin-Layer Foods Based on Two Study Cases. Arab. J. Sci. Eng..

[35] Prudhvi P.V.V.P., Deepika S., Sutar P.P. (2022). Modeling moisture and solids transfer kinetics during a novel microwave assisted water absorption-desorption process of dry red gram (*Cajanus cajan* L.) splits. J. Food Eng..

[36] Da Silva W.P., De Lima A.G., Pereira J.C., Gomes J.P., Queiroz A.J., De Figueirêdo R.M., Paiva Y.F., Dos Santos F.S., De Melo B.A., Moura H.V. (2024). A Diffusion Model to Describe Water Absorption by Red Rice during Soaking: Variable Mass Diffusivity, Variable Volume, Use of Boundary-Fitted Coordinates. Processes.

[37] Adduci G., Petrosino F., Manoli E., Cardaropoli E., Coppola G., Curcio S. (2024). Transport phenomena in pasta drying: A dough-air double domain advanced modeling. J. Food Eng..

[38] Ge M., Chen G., Liu W., Liu C. (2024). Study of heat and mass transfer during drying process of maize grain pile based on computed tomography. Biosyst. Eng..

[39] Wang X., Zhou Y., Shi Y., Wang Q., Hui Y., Ding H. (2025). Permeability prediction of bulk wheat for storage based on micro-computed tomography and lattice Boltzmann method. Biosyst. Eng..

[40] Datta A.K. (2007). Porous media approaches to studying simultaneous heat and mass transfer in food processes. I: Problem formulations. J. Food Eng..

[41] Claessens B., Hitsov I., Verliefde A., Nopens I. (2022). Analyzing transport in ceramic membranes for organic solvent nanofiltration using Maxwell-Stefan theory. Chem. Eng. Sci..

[42] Costa-Corredor A., Pakowski Z., Lenczewski T., Gou P. (2010). Simulation of simultaneous water and salt diffusion in dry fermented sausages by the Stefan–Maxwell equation. J. Food Eng..

[43] Wu S., Wang J., Zhang L., Liu S., Li C. (2024). Effects of Osmotic Dehydration on Mass Transfer of Tender Coconut Kernel. Foods.

[44] Vitrac O., Nguyen P.-M., Hayert M. (2022). In Silico Prediction of Food Properties: A Multiscale Perspective. Front. Chem. Eng..

[45] Ajani C.K., Zhu Z., Sun D.-W. (2023). Microscale Modelling of Flow, Heat and Mass Transport During Vacuum Cooling of Porous Foods: Effective Property Computation. Transp. Porous Media.

[46] Li P., Ma C., Chen Z., Wang H., Wang Y., Bai H. (2023). A Review: Study on the Enhancement Mechanism of Heat and Moisture Transfer in Deformable Porous Media. Processes.

[47] Van Der Sman R.G.M. (2022). MULTICUBED: Multiscale-multiphysics simulation of food processing. Food Struct..

[48] Golpour I., Guiné R.P.F., Poncet S., Golpour H., Amiri Chayjan R., Amiri Parian J. (2021). Evaluating the heat and mass transfer effective coefficients during the convective drying process of paddy (*Oryza sativa* L.). J. Food Process Eng..

[49] Niu X.-X., Deng L.-Z., Wang H., Wang Q.-H., Xu M.-Q., Li S.-B., Okaiyeto S.A., Xiao H.-W. (2024). Transformation of cell wall pectin profile during postharvest ripening process alters drying behavior and regulates the sugar content of dried plums. Food Chem..

[50] Gautam S., Kathuria D., Hamid, Dobhal A., Singh N. (2024). Vacuum impregnation: Effect on food quality, application and use of novel techniques for improving its efficiency. Food Chem..

[51] Welsh Z.G., Simpson M.J., Khan M.I.H., Karim M.A. (2023). Generalized moisture diffusivity for food drying through multiscale modeling. J. Food Eng..

[52] Martínez Vera C., Vizcarra Mendoza M.G. (2022). Concentration-dependent moisture diffusion coefficient estimation in peas drying considering shrinkage: An observer approach. Biosyst. Eng..

[53] Nemati R., Takhar P.S. (2025). Microstructural characterization of a wheat-based food material using image analysis and pore network modeling during baking. J. Food Sci..

[54] Dadmohammadi Y., Kantzas A., Yu X., Datta A.K. (2020). Estimating permeability and porosity of plant tissues: Evolution from raw to the processed states of potato. J. Food Eng..

[55] Aghajanzadeh S., Sultana A., Mohammad Ziaiifar A., Khalloufi S. (2024). Formation of pores and bubbles and their impacts on the quality attributes of processed foods: A review. Food Res. Int..

[56] Sánchez-Torres E.A., Giacomozzi A.S., Abril B., Benedito J., Bon J., García-Pérez J.V. (2025). Analysis of the Induced Mild Heating by Airborne Ultrasound Application on the Convective Drying of Pork Liver. Food Bioprocess Technol..

[57] Wang J., Chen Y., Wang H., Wang S., Lin Z., Zhao L., Xu H. (2022). Ethanol and blanching pretreatments change the moisture transfer and physicochemical properties of apple slices via microstructure and cell-wall polysaccharides nanostructure modification. Food Chem..

[58] Zhang C., Lyu X., Zhao W., Yan W., Wang M., Kuan Rei N.G., Yang R. (2021). Effects of combined pulsed electric field and blanching pretreatment on the physiochemical properties of French fries. Innov. Food Sci. Emerg. Technol..

[59] Shorstkii I., Sosnin M., Smetana S., Toepfl S., Parniakov O., Wiktor A. (2022). Correlation of the cell disintegration index with Luikov’s heat and mass transfer parameters for drying of pulsed electric field (PEF) pretreated plant materials. J. Food Eng..

[60] Shi Y., Wang Y., Shi J., Li Z., Huang X., Liang J., Zhang X., Zhang D., Zou X., Hu X. (2023). Simulation of diffusion behavior of NaCl in multi-tissue beef marination process. Food Chem..

[61] Gouyo T., Goujot D., Bohuon P., Courtois F. (2021). Multi-compartment model for heat and mass transfer during the frying of frozen pre-fried French fries. J. Food Eng..

[62] Welsh Z.G., Simpson M.J., Khan M.I.H., Karim A. (2021). A multiscale approach to estimate the cellular diffusivity during food drying. Biosyst. Eng..

[63] Guo Y., Gao J., Bai Y., Wang X., Xu X., Lu X., Yue J., Han M. (2024). Effect of pulsed electric field (PEF) on NaCl diffusion in beef and consequence on meat quality. Meat Sci..

[64] Aguirre-García M., Cortés-Zavaleta O., Ruiz-Espinosa H., Ochoa-Velasco C.E., Ruiz-López I.I. (2022). The role of coupled water and solute diffusion and product shrinkage during osmotic dehydration. J. Food Eng..

[65] Dehghannya J., Habibi-Ghods M. (2025). Computer simulation of microwave-assisted drying: Coupled influence of microwave power and pulse ratio on product and process characteristics. Curr. Res. Food Sci..

[66] Purlis E. (2019). Modelling convective drying of foods: A multiphase porous media model considering heat of sorption. J. Food Eng..

[67] Rani P., Tripathy P.P. (2023). CFD coupled heat and mass transfer simulation of pineapple drying process using mixed-mode solar dryers integrated with flat plate and finned collector. Renew. Energy.

[68] Joardder M.U.H., Karim A. (2025). Dynamic Temperature-Responsive MW Pulsing for Uniform and Energy-Efficient Plant-Based Food Drying. Energies.

[69] Oladejo A.O., Gruber S., Foerst P. (2025). Applications of non-invasive measuring techniques of internal changes during drying of food products. J. Food Eng..

[70] Teleken J.T., Amorim S.M., Rodrigues S.S.S., De Souza T.W.P., Ferreira J.P., Carciofi B.a.M. (2025). Heat and Mass Transfer in Shrimp Hot-Air Drying: Experimental Evaluation and Numerical Simulation. Foods.

[71] Zhao J., Qin F., Kang Q., Derome D., Carmeliet J. (2021). Pore-scale simulation of drying in porous media using a hybrid lattice Boltzmann: Pore network model. Dry. Technol..

[72] Batuwatta-Gamage C.P., Rathnayaka C., Karunasena H.C.P., Jeong H., Karim A., Gu Y.T. (2023). A novel physics-informed neural networks approach (PINN-MT) to solve mass transfer in plant cells during drying. Biosyst. Eng..

[73] Sakin-Yilmazer M., Kaymak-Ertekin F., Ilicali C. (2012). Modeling of simultaneous heat and mass transfer during convective oven ring cake baking. J. Food Eng..

[74] Pacheco Plata F., Gutiérrez Dorado R., Iribe Salazar R., Carrazco Escalante M., Caro Hernández O., Camacho Hernández L., Vázquez López Y., Cronin K., Caro Corrales J. (2024). Modeling of moisture content during baking with different approaches for effective diffusivity and evaluation of quality variables in baked potato slices. J. Food Sci..

[75] Reddy R.S., Arepally D., Datta A.K. (2022). Inverse problems in food engineering: A review. J. Food Eng..

[76] Zhang R., Li F., Tang J., Koral T., Jiao Y. (2020). Improved accuracy of radio frequency (RF) heating simulations using 3D scanning techniques for irregular-shape food. LWT.

[77] Zheng Z., Ren L., Xie W., Wei S., Fu H., Yang P., Xu J., Yang D. (2024). Drying stress analysis and cracking prediction of the component of maize based on viscoelastic stress-strain model. Innov. Food Sci. Emerg. Technol..

[78] Li W., Shi Y., Huang X., Li Z., Zhang X., Zou X., Hu X., Shi J. (2024). Study on the Diffusion and Optimization of Sucrose in Gaido Seak Based on Finite Element Analysis and Hyperspectral Imaging Technology. Foods.

[79] Anders A., Choszcz D., Markowski P., Lipiński A.J., Kaliniewicz Z., Ślesicka E. (2019). Numerical Modeling of the Shape of Agricultural Products on the Example of Cucumber Fruits. Sustainability.

[80] Zennoune A., Latil P., Ndoye F.-T., Flin F., Perrin J., Geindreau C., Benkhelifa H. (2021). 3D Characterization of Sponge Cake as Affected by Freezing Conditions Using Synchrotron X-ray Microtomography at Negative Temperature. Foods.

[81] Yu Y., Jia C., Wang J., Pi F., Dai H., Liu X. (2023). Characterizing the Internal Structure of Chinese Steamed Bread during Storage for Quality Evaluation Using X-ray Computer Tomography. Sensors.

[82] Zare D., Akbarzadeh S., Nematollahi M.A., Loghavi M. (2020). Simulation of hot air infrared-assisted green peas drying using finite element method. J. Food Process Eng..

[83] Silva Júnior M.a.V., Leite M.A., Dacanal G.C. (2023). Modelling of convective drying of potatoes polyhedrons. Int. J. Food Eng..

[84] Das R., Prasad K. (2024). Finite element modeling in heat and mass transfer of potato slice dehydration, nonisotropic shrinkage kinetics using arbitrary Lagrangian–Eulerian algorithm and artificial neural network. J. Food Process Eng..

[85] Al Faruq A., Farahnaky A., Dokouhaki M., Khatun H.A., Trujillo F.J., Majzoobi M. (2025). Technological Innovations in Freeze Drying: Enhancing Efficiency, Sustainability, and Food Quality. Food Eng. Rev..

[86] Cevoli C., Panarese V., Catalogne C., Fabbri A. (2020). Estimation of the effective moisture diffusivity in cake baking by the inversion of a finite element model. J. Food Eng..

[87] Buzrul S. (2022). Reassessment of Thin-Layer Drying Models for Foods: A Critical Short Communication. Processes.

[88] Iribe-Salazar R., Caro-Corrales J., Vázquez-López Y. (2021). Analysis of random variability in Tortilla shells baking. J. Food Eng..

[89] Rurush E., Alvarado M., Palacios P., Flores Y., Rojas M.L., Miano A.C. (2022). Drying kinetics of blueberry pulp and mass transfer parameters: Effect of hot air and refractance window drying at different temperatures. J. Food Eng..

[90] Moradi Maryamnegari S., Ashrafizadeh A., Baake E., Guglielmi M. (2023). Effects of thermal boundary conditions on the performance of spray dryers. J. Food Eng..

[91] González-Camacho M., Iribe-Salazar R., Vázquez-López Y., Carrazco-Escalante M., Caro-Hernández O., Gil-Gaxiola M., Gutiérrez-Dorado R., Cronin K., Caro-Corrales J. (2026). Modelling of moisture content during baking of beetroot slices via Fick’s law: A comparison of constant and variable effective diffusivity. J. Food Eng..

[92] Wang N., Kan A., Mao S., Huang Z., Li F. (2021). Study on heat and mass transfer of sugarcane stem during vacuum pre-cooling. J. Food Eng..

[93] Hassan A., Joardder M.U.H., Karim A. (2025). A CFD integrated drying model for improving drying conditions in industry Scale dryers. Therm. Sci. Eng. Prog..

[94] Sourya D.P., Panda D., Kharaghani A., Tsotsas E., Gurugubelli P.S., Surasani V.K. (2023). Lattice Boltzmann simulations for the drying of porous media with gas–side convection–diffusion boundary. Phys. Fluids.

[95] Yin J., Guo M., Liu G., Ma Y., Chen S., Jia L., Liu M. (2022). Research Progress in Simultaneous Heat and Mass Transfer of Fruits and Vegetables During Precooling. Food Eng. Rev..

[96] Shahari N., Hasnan H.A., Hanan A.Y., Noor Ishak N.a.H. (2019). Analysis of two-dimensional (2D) Fruit Drying Process through Heat and Mass Transfer Model. IOP Conf. Ser. Mater. Sci. Eng..

[97] Van Der Sman R.G.M. (2023). Lattice Boltzmann model for freezing of French fries. Curr. Res. Food Sci..

[98] Beltran J., Mayorga E., Jalabe G., Moreno F. (2025). Mathematical modelling of freezing of vacuum-packed beef with irregular geometries and structures. Int. J. Refrig..

[99] Chokngamvong S., Suvanjumrat C. (2024). Development of conjugate heat- and moisture-transfer model for pineapple drying using OpenFOAM. Case Stud. Therm. Eng..

[100] Chen X., Liu Y., Zhang R., Zhu H., Li F., Yang D., Jiao Y. (2022). Radio Frequency Drying Behavior in Porous Media: A Case Study of Potato Cube with Computer Modeling. Foods.

[101] Ramírez-Rivera M.J., Díaz-Ovalle C.O., Ramos-Ojeda E., Castrejón-González E.O. (2024). CFD simulation analysis of fouling formation in a milk falling-film evaporator. Food Bioprod. Process..

[102] Joshi A., Pratihar A.K. (2023). Experimental and simulation studies on blanching and its impact on the drying rate of carrot. J. Food Process Eng..

[103] Kapil A., Wombwell C., Kegel L.L., Hamlin M.D. (2025). Model-aided process development for scalable spray drying of sticky substances. Front. Chem. Eng..

[104] Maidannyk V.A., Simonov Y., Mccarthy N.A., Ho Q.T. (2024). Water Effective Diffusion Coefficient in Dairy Powder Calculated by Digital Image Processing and Through Machine Learning Algorithms of CLSM Micrographs. Foods.

[105] Carrillo Luis V., Beristain Rios D., Hernández-Flores O.A., Romero-Salazar C., Sandoval-Torres S. (2024). Mathematical Modeling of Goat Meat Drying Kinetics with Thermal Oscillations. Foods.

[106] Guo J., Zhang X., Liu Y., Wu J., Xu H., Xiao H., Ai Z., Gong P. (2026). Intelligent monitoring, predicting, and control technology of food drying: Recent advances, challenges, and future prospects. Food Control.

[107] Mansour Y., Rouaud O., Slim R., Rahmé P. (2024). Thermal characterization of a high-temperature industrial bread-baking oven: A comprehensive experimental and numerical study. Appl. Therm. Eng..

[108] Chakraborty S., Dash K.K. (2023). A comprehensive review on heat and mass transfer simulation and measurement module during the baking process. Appl. Food Res..

[109] Do T.C., Le Q.T., Tran T.T. (2024). Modeling for Apple-Slice Drying in Carbon Dioxide Gas. Agriculture.

[110] Ajani C.K., Zhu Z., Sun D. (2023). Shrinkage during vacuum cooling of porous foods: Conjugate mechanistic modelling and experimental validation. J. Food Eng..

[111] Ghaderi A., Dehghannya J., Ghanbarzadeh B. (2022). Multiphase flow, heat and mass transfer modeling during frying of potato: Effect of food sample to oil ratio. Int. J. Food Eng..

[112] Ali I., Saleem M.T. (2023). Applications of Orthogonal Polynomials in Simulations of Mass Transfer Diffusion Equation Arising in Food Engineering. Symmetry.

[113] Park H.W., Yoon W.B. (2018). Development of a Novel Image Analysis Technique to Detect the Moisture Diffusion of Soybeans [*Glycine max* (L.)] During Rehydration Using a Mass Transfer Simulation Model. Food Bioprocess Technol..

[114] Li Y., Liang M., Li J., Jiang K., Li X., Zheng Z. (2025). Simulation and Experimental Studies of Heat-Mass Transfer and Stress–Strain in Carrots During Hot Air Drying. Agriculture.

[115] Tegenaw P.D., Verboven P., Vanierschot M. (2022). Numerical and experimental study of airflow resistance across an array of sliced food items during drying. J. Food Eng..

[116] Batuwatta-Gamage C.P., Rathnayaka C.M., Karunasena H.C.P., Wijerathne W.D.C.C., Jeong H., Welsh Z.G., Karim M.A., Gu Y.T. (2022). A physics-informed neural network-based surrogate framework to predict moisture concentration and shrinkage of a plant cell during drying. J. Food Eng..

[117] Cao S., Yang C., Zang Y., Li Y., Gu J., Ding H., Yao X., Zhu R., Wang Q., Dong W. (2023). Simulated and Verification of Mass and Heat Transfer Coupled Model of Jujube Slices Dried by Hot Air Combined with Radio Frequency Heat Treatment at Different Drying Stages. Foods.

[118] Chen P., Chen N., Zhu W., Wang D., Jiang M., Qu C., Li Y., Zou Z. (2023). A Heat and Mass Transfer Model of Peanut Convective Drying Based on a Two-Component Structure. Foods.

[119] Seranthian K., Datta A. (2023). Dynamics of cupcake baking: Coupled multiphase heat and mass transport in a deformable porous material. Chem. Eng. Sci..

[120] Dehghannya J., Ghaderi A., Ghanbarzadeh B. (2025). Three-dimensional modeling of coupled momentum, heat, and mass transfer during potato frying: Effects of oil temperature, type, frying load, and fryer heating cycles. Curr. Res. Food Sci..

[121] Yang W., Long L., Zhang L., Xu K., Huang Z., Ye H. (2025). Heat and mass transfer and deformation during chiffon cake baking. J. Food Eng..

[122] Seranthian K., Datta A., Clanton A. (2024). Ingredient functionality in batter-type cake baking: Coupled multiphase poro-hygro-viscoelastic model. J. Food Eng..

[123] Dehghannya J., Ngadi M. (2023). The application of pretreatments for producing low-fat fried foods: A review. Trends Food Sci. Technol..

[124] Tena J., Fueyo N. (2026). A Computational Fluid Dynamics model for predicting food browning through melanoidin kinetics during baking. J. Food Eng..

[125] Payne M.R., Morison K.R. (1999). A multi-component approach to salt and water diffusion in cheese. Int. Dairy J..

[126] Yang H., Min S., Yang J., Lee M., Park S., Eun J., Chung Y. (2024). Predictive modeling and mass transfer kinetics of tumbling-assisted dry salting of kimchi cabbage. J. Food Eng..

[127] Dutta A., Subramanian A.S., Chakraborty R., Erdogdu F. (2020). Numerical modeling of water uptake in white rice (*Oryza sativa* L.) using variable diffusivity approach. Biosyst. Eng..

[128] Zhang J., Zhao F., Li C., Ban X., Gu Z., Li Z. (2024). Acceleration mechanism of the rehydration process of dried rice noodles by the porous structure. Food Chem..

[129] Sam Saguy I., Marabi A., Wallach R. (2005). New approach to model rehydration of dry food particulates utilizing principles of liquid transport in porous media. Trends Food Sci. Technol..

[130] Van Der Sman R.G.M., Vergeldt F.J., Van As H., Van Dalen G., Voda A., Van Duynhoven J.P.M. (2014). Multiphysics pore-scale model for the rehydration of porous foods. Innov. Food Sci. Emerg. Technol..

[131] Nugrahedi P.Y., Soesilo S.A., Perdana J., Yudiar H., Sanyoto G.J. (2025). Moisture Migration and Its Prevention in Multi-Domain Bakery Products: A Review. Food Rev. Int..

[132] Zardetto S., Martello A.D., Pasini G. (2025). Moisture migration model of packed fresh-filled pasta during storage under different humidity conditions. Innov. Food Sci. Emerg. Technol..

[133] Shetty H., Patel B., Saibene D., Vodovotz Y., Campanella O.H. (2025). Predicting equilibrium water activity using different moisture isotherms and estimating moisture transfer in a multicomponent mixture for vegetable chips. LWT.

[134] Linnenkugel S., Paterson A.H.J., Huffman L.M., Bronlund J.E. (2022). Prediction of the effect of water on the glass transition temperature of low molecular weight and polysaccharide mixtures. Food Hydrocoll..

[135] Xie Y., Jin X., Bi J. (2025). Enhanced freeze-drying efficiency in restructured peach: Multiscale insights into heat and mass transfer mechanisms from experiments and computational simulations. Food Res. Int..

[136] Kannapinn M., Dorer D., Schäfer M., Weeger O. (2026). Digital twins for autonomous thermal food processing: A model predictive control study with reduced-order models of augmented neural ordinary differential equation type. J. Food Eng..

[137] Raissi M., Perdikaris P., Karniadakis G.E. (2019). Physics-informed neural networks: A deep learning framework for solving forward and inverse problems involving nonlinear partial differential equations. J. Comput. Phys..

